# Dynamic changes in microbiota and mycobiota during spontaneous ‘Vino Santo Trentino’ fermentation

**DOI:** 10.1111/1751-7915.12337

**Published:** 2016-01-18

**Authors:** Irene Stefanini, Davide Albanese, Agostino Cavazza, Elena Franciosi, Carlotta De Filippo, Claudio Donati, Duccio Cavalieri

**Affiliations:** ^1^ Department of Computational BiologyResearch and Innovation CentreFondazione Edmund Mach (FEM)San Michele all'AdigeItaly; ^2^ Food Quality Nutrition & Health DepartmentResearch and Innovation CentreFondazione Edmund Mach (FEM)San Michele all'AdigeItaly; ^3^ Institute of Biometeorology – IBIMETNational Research CouncilFlorenceItaly; ^4^ Department of BiologyUniversity of FlorenceFlorenceItaly

## Abstract

Vino Santo is a sweet wine produced from late harvesting and pressing of Nosiola grapes in a small, well‐defined geographical area in the Italian Alps. We used metagenomics to characterize the dynamics of microbial communities in the products of three wineries, resulting from spontaneous fermentation with almost the same timing and procedure. Comparing fermentation dynamics and grape microbial composition, we show a rapid increase in a small number of wine yeast species, with a parallel decrease in complexity. Despite the application of similar protocols, slight changes in the procedures led to significant differences in the microbiota in the three cases of fermentation: (i) fungal content of the must varied significantly in the different wineries, (ii) *P* *ichia membranifaciens* persisted in only one of the wineries, (iii) one fermentation was characterized by the balanced presence of *S* *accharomyces cerevisiae* and *H* *anseniaspora osmophila* during the later phases. We suggest the existence of a highly winery‐specific ‘microbial‐terroir’ contributing significantly to the final product rather than a regional ‘terroir’. Analysis of changes in abundance during fermentation showed evident correlations between different species, suggesting that fermentation is the result of a continuum of interaction between different species and physical–chemical parameters.

## Introduction

In oenology, the quality, taste and style of wine are traditionally considered to be influenced by the cultivar of the fermented grapes, geographical factors such as the vineyard mesoclimate, topoclimate and microclimate, soil geology and pedology, and the agronomic approach used (Van Leeuwen & Seguin, 2006). Recently, the microbial population involved in the fermentation process has been shown to play a pre‐eminent role in determining the properties of wine (Carreto *et al*., 2008; Renouf *et al*., 2002) through the production of secondary metabolites that contribute to wine aromas (Calabretti *et al*., 2012). In a recent study, Knight and colleagues (2015) experimentally demonstrated that the wine organoleptic characteristics are affected by the origin and genetics of *Saccharomyces cerevisiae* natural strains, providing objective evidence for a microbial aspect to *terroir*. Several studies have shown that the microbial population present at the moment of grape harvesting and during early fermentation belongs to the vineyard (Polsinelli *et al*., 1996; Barata *et al*., 2012), providing an alternative mechanism through which the environment may influence wine quality and taste (Lopes *et al*., 2002; Renouf *et al*., 2002; Carreto *et al*., 2008). The advent of culture‐independent methods based on high‐throughput sequencing has shown the high level of variability of leaf‐associated microbial communities in *Vitis vinifera* (Pinto *et al*., 2014) and geographical structuring of the mycobiota associated with the surface of grapes immediately before harvest (Taylor *et al*., 2014). The geographical area, together with cultivar, climate and vintage, has been shown to be the major determinant for the microbiota of must at the beginning of the fermentation process (Bokulich *et al*., 2014). Despite the fact that a part of this early stage microbiota does not survive the stressful conditions of late must fermentation, it still plays a role in shaping the entire process (Heard and Fleet, 1988).

A few yeasts (among which, *S. cerevisiae*) have a dominating influence in must fermentation because of their ability to conduct alcoholic fermentation, but some moulds and bacteria can still contribute to the overall process (Fleet, 1993; Fugelsang and Edwards, 2007; Verginer *et al*., 2010). Fine control of the composition of must microbiota is of paramount importance for the quality of the final product, since different components of must microbiota can contribute in contrasting ways to the aroma of the final product, giving either pleasant or undesirable aromatic notes to the wine. During fermentation, the microbiota can be affected by both microbial and chemical–physical factors. Some fungal species can either carry out antimicrobial activity against certain other species/strains (Oro *et al*., 2014) or have a positive effect on the growth of other species (Contreras *et al*., 2014). On the other hand, although the mechanisms are still not fully described, it is well known that several factors related to grape juice (i.e. grape composition and chemical characteristics, ethanol accumulation and temperature) can affect the kinetics of yeast growth (Fleet and Heard, 1993; Bisson, 1999; Zott *et al*., 2008). To avoid the production of undesirable metabolites during must fermentation, the routine of inoculating wine fermentations with pure *S. cerevisiae* cultures was introduced by Müller‐Thurgau in 1890 (Calabretti *et al*., 2012; Pretorius, 2000) and is now widely used. Nevertheless, the indigenous microbiota deriving from grapes contributes to fermentation even in the case of inoculation of selected *S. cerevisiae* strains (Fleet 1993). Recently, winemakers have started to realize the potential contribution offered by the indigenous microbial population in producing a wine closely associated with geographical origin (Tristezza *et al*., 2013). The studies carried out so far have described the microbial population either following a limited number of strains and species at selected time points, or using metagenomics to compare the wine microbiota before or after fermentation, losing the dynamic process of microbial populations. The evolution of must microbiota during fermentation, how the species present on the grapes and in the early fermentation stage influence late stage fermentation and how this evolution is influenced by chemical–physical parameters of the fermentation is not known in detail.

In this work, we used high‐throughput amplicon sequencing to study the dynamics of the natural microbial populations (both bacteria and fungi) associated with the fermentation of Vino Santo. Vino Santo Trentino is a sweet wine made with Nosiola, a grape variety grown almost exclusively in Trentino and in particular in the ‘Valle dei Laghi’ (literally: valley of the lakes). Looser grapes are harvested progressively when they ripen. Traditionally, grapes are crushed during Holy Week (settimana santa), before Easter Sunday. Given the strict adherence to traditional fermentation techniques and the use of a single cultivar (Nosiola), Vino Santo fermentation represents an excellent opportunity to identify the connections between the fermentation process and the microbiota. The peculiarity of Vino Santo fermentation is that, as for other sweet wine fermentation, the grapes are subjected to extended ripening before harvesting and to a prolonged period of drying before crushing, which enhances the abundance of *Botrytis cinerea*, a mould often found on the skin of grapes used for sweet wine production and known to have an impact on microbiota composition (Joyeux *et al*., 1984; Mills *et al*., 2002; Nisiotou *et al*., 2007; Bokulich *et al*., 2014). In addition to triggering secondary effects through enrichment in *Botrytis*, the extended ripening and the long drying period also make the chemical–physical characteristics of the must more extreme than those of must obtained from fresh grapes, the most evident factor being the high sugar content of must, resulting in a selection of osmotolerant species (Tofalo *et al*., 2009). In addition, single‐variety vinification prevents the deviation of microbial populations shown to be associated with the vine cultivar (Bokulich *et al*., 2014). After drying, the grapes are crushed and fermentation starts. Vino Santo is produced in small amounts in a period in which no other fermentation is carried out in the cellars. This allows us to presume that environmental contamination is limited to resident microbiota, i.e. the species associated with dried grapes and those permanently associated with the cellar environment, while commercial starter cultures, which are sometimes used during vintage period of winemaking, were not present. Moreover, no blending with other wines takes place, as the characteristics of Vino Santo are unique. The limited geographical production area, the unique winemaking technique and the unusual period in which it is fermented, make this type of vinification an excellent model for studying the links between microbiota, wineries and the area. Fermentation occurs in stainless steel tanks and takes place in spring and summer, and stops naturally when the ethanol concentration is around 12–15 degrees. The wine is then racked and transferred into wood barrels, usually 228 L barriques, where it is aged for 4 to 10 years.

## Results

### Sampling and experimental strategy

**Figure 1:**
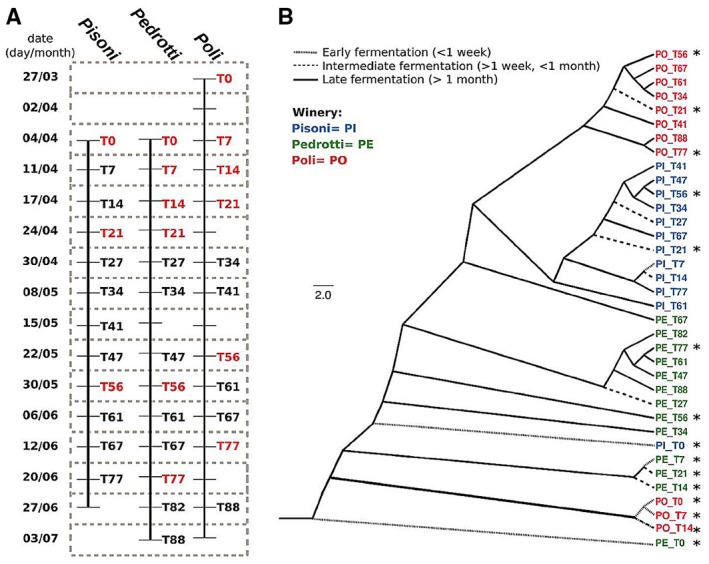
Sample details and selection. A. Must samples were collected from the three wineries (Poli, Pedrotti and Pisoni) once a week every week from the beginning of fermentation until completion, occurring on the 88th day of fermentation at all the wineries. Samples were named with ‘T’ (time) and the number of days passing from the beginning of fermentation (0–88). Each sample was analysed using PCR‐RFLP (see details in *materials and methods*), and the samples most representative of each winery (see clustering in different parts of the dendrogram in B) were selected for meta‐taxonomic analysi**s** (highlighted in red). B. Dendrograms were built on distances calculated on the ITS1‐5.8S–ITS2 regions PCR‐RFLP profiles. Amplified ITS1‐5.8S–ITS2 regions were digested with both *HaeIII* and *HinfI* enzymes. The most parsimonious trees were found on 1/0 (presence/ absence) band profiles with p enny software (p hylip). Finally, a consensus dendrogram was obtained with consense (p hylip) and drawn with figtree. Label colours: red = Poli winery must samples, blue = Pisoni winery must samples, green = Pedrotti winery must samples. Asterisks indicate samples that were selected for meta‐taxonomic analysis by 454‐pyrosequencing.

To examine the dynamic changes in microbial populations driving must fermentation, three Vino Santo products were investigated from the beginning to the end of fermentation (3 months), at the Poli, Pisoni and Pedrotti wineries located in the Valle dei Laghi in Trentino, Italy. The three wineries followed almost the same procedure to carry out Vino Santo fermentation (see further details in the *Supporting information*). Samples were collected weekly from each winery (at least 12 samples for each winery) and named with the number of days passing from the beginning of fermentation (T0–T88; Fig. 1A and Table S1).

To obtain a preliminary indication of the similarities between the mycobiota in the different samples and select representative samples for high‐throughput amplicon sequencing using Next Generation Sequencing (NGS), whole microbial deoxyribonucleic acid (DNAs) extracted from the samples were initially explored by means of fungal polymerase chain reaction restriction fragment length polymorphism (PCR‐RFLP; ITS1‐5.8S‐ITS2 regions treated with the *HaeIII* and *HinfI* restriction enzymes separately). The PCR‐RFLP profiles were then compared and based on their clustering (Fig. 1B). Meta‐taxonomic analysis was performed by using 454‐pyrosequencing (primers are listed in Table S2) on the most representative samples (3 for the Pedrotti winery, 6 for the Pisoni and Poli wineries). While the meta‐taxonomic approach allows definition of the relative composition of microbial populations, in a dynamic environment (such as must), determination of absolute operational taxonomic unit (OTU) abundances is pivotal for correct comparison of different populations. For this reason, the total amount of microbial (fungal or bacterial) DNA was quantified for each sample, and absolute microbial amounts were calculated. Moreover, to understand the impact of the fungal population on the dynamics of the bacterial component of the microbiota, we also investigated the composition of the bacterial populations present in the must samples of one winery showing a particular fungal pattern at the end of the fermentation.

### PCR‐RFLP profiles of fungal populations highlight sample differentiation between wineries and the fermentation stages

For preliminary screening of the dynamic variability of the fungal component of the microbiota, we performed PCR‐RFLP on the fungal ribosomal intergenic region ITS1‐5.8S‐ITS2 amplified from the DNA extracted from must samples. Hierarchical clustering of the resulting PCR‐RFLP patterns showed that the composition of the mycobiota changed during the first month, after which it assumed a composition characteristic of the winery, which was maintained until the end of fermentation (Fig. 1B). Moreover, the Pedrotti and Poli samples collected in the first month of the process clustered separately from the rest of the samples collected later in the same wineries, but were still grouped according to their origin (Pedrotti: T7, T14, T21; Poli: T0, T14, T21; Fig. 1B). Of the must samples from the Pisoni winery, only the first time point (PI_T0) clustered separately from the rest of samples collected at the same winery.

Using the clustering results, we selected between three and six representative samples per winery according to the following criteria: (i) the PCR‐RFLP profiles differed from other samples selected from the same winery; (ii) they were representative of the whole duration of fermentation. The fungal populations changed during the initial phases of the fermentation, and, as expected, they became stable at late phases of fermentation (as indicated by the clustering of the late samples; Fig. 1B). With this in mind, six Poli and Pisoni must samples (T0, T7, T14, T21, T56, T77) and three Pedrotti must samples (T0, T21, T56) were selected (marked by the asterisks in Fig. 1B) to determine the absolute amount of total fungal content using quantitative Real Time PCR (qRT‐PCR) and meta‐taxonomics analysis of the mycobiota.

### Dynamics of the Vino Santo fungal population

**Figure 2:**
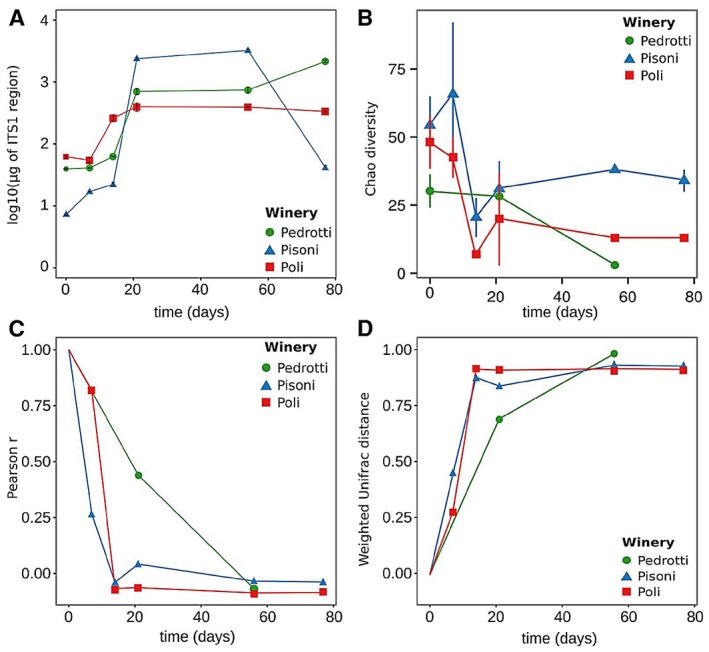
Fermentation dynamics. A. Fungal DNA quantification. Total fungal DNA was quantified through qRT‐PCR by using universal primers amplifying the ITS1 region. Standard curves were constructed using PCR products of the ITS1 rDNA of a metagenomic sample. Error bars: standard deviations of three technical replicates. B. Chao1 estimator of the number of fungal OTUs observed in the samples using meta‐taxonomic analysis. C. Pearson correlation r of fungal populations at T0 against each other at time points. D. Weighted u nifrac distances of fungal population at T0 with respect to fungal populations at the other time points. Lines represent only guidelines.

It is well known that fungal population size (in terms of colony forming units) changes widely during fermentation (Maturano *et al*., 2015). Thus, we measured the total fungal DNA content in each sample by means of qRT‐PCR (Fig. 2A) of the ITS1 region using universal primers. We found that, after an initial increase, the amount of fungal DNA reached a plateau during each fermentation. The time at which this occurred varied in the different wineries, with the Pedrotti and Pisoni wineries reaching the plateau at T21, and the Poli fermentation being faster, as it had already reached the plateau at T14 (Fig. 1A). After T54, while the amount of fungi in the Pisoni fermentation drastically decreased, fungal population abundance remained unchanged during the Poli fermentation and increased slightly during the Pedrotti fermentation.

To obtain a detailed description of the microbial population and to delve into the dynamics of the microbial species during fermentation, the samples previously selected through PCR‐RFLP analysis (Fig. 1B) and subjected to fungal DNA quantification (Fig. 2A) were analysed through meta‐taxonomics analysis by means of 454‐pyrosequencing, using primers specific for the ITS1‐5.8S–ITS2 fungal DNA region (for a complete list of taxonomic assignments refer to Table S3). To measure the alpha (within sample) diversity of the fungal populations driving the fermentation, we measured the Chao1 estimator of (Fig. 2B) the number of OTUs present in the samples, the measured number of OTUs and the Shannon entropy (Fig. S1A). In the case of both Pisoni and Poli fermentation, we found a sharp decrease in alpha diversity in the first 2 weeks, followed by a plateau. The decrease in alpha diversity during the Pedrotti fermentation, although detectable, was less marked.

Comparison of these results with the total fungal DNA quantification (Fig. 2A) showed that this decrease in alpha diversity coincided with an increase in the total fungal component of the microbiota. While the fungal population was initially small (as indicated by the low amount of fungal DNA; Fig. 2A) but made up of a large number of different species (high alpha diversity; Fig. 2B and S1A), it became larger but more simplified after the first 2 weeks of fermentation, ultimately resulting in a population dominated by a relatively low number of species.

To explore to what extent the dynamics of the fungal population were determined by the initial structure of the microbiota and whether the dominating species in the early stages influenced the fermentation at later stages, we calculated the Pearson correlation coefficient between the population structure at the initial time (T0) and each of the later time points (Fig. 2C). The data showed that in the cases of both Pisoni and Poli fermentation after 2 weeks (T14), the structures of the mycobiota were already uncorrelated with their initial state. Again, this timescale corresponded to the time of the initial increase in the total fungal component of the microbiota shown in Fig. 2A and to the decrease in alpha diversity. Similarly, fast differentiation of mycobiota from its composition at time T0 was highlighted by measuring the weighted unifrac distance (Lozupone *et al*., 2013) between the samples' microbiota at time T0 and at later times (Fig. 2D). Given that the unifrac distance weights the variation in relative abundance with the genetic relatedness of the OTUs, this result demonstrates a large change in the taxonomic structure of the two fermentations. The dynamics of the Pedrotti fermentation, for which we had fewer data points, was much slower. However, in this case, the situation was again similar to the other two fermentations after T57.

These indications, seen in the light of the results for fungal DNA quantification, suggest that the natural fungal population present in fresh musts have initially driven the fermentation with only a small increase in cell number (the fungal DNA content at T0 and T07 changed only slightly in all the fermentations; Fig. 2A). The total fungal DNA content then increased due to the increase in a few species that were present at low relative abundance at the beginning of fermentation, simplifying the structure of the population (as shown by the decrease in alpha diversity; Fig. 2B and Fig. S1A), quickly differentiating the fungal population from the initial one (Fig. 2C and D).

### Composition of the fungal populations during Vino Santo fermentation

**Figure 3:**
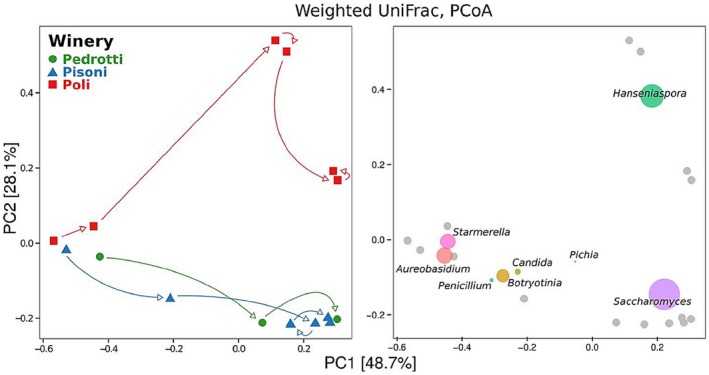
Sample fungal population diversity. Distribution of variables over the first two components of the PCoA carried out on weighted u ni f rac distances. Left: first two coordinates of the PCoA. Arrows connect consecutive time points. Right: the same PCoA plot with the eight most abundant OTUs (showing the highest average relative abundance across all the samples) overlaid as coloured points with a size proportional to the mean relative abundance of the taxon across all samples. Genus coordinates were calculated as the weighted average across sample coordinates. Grey dots in the right plot represent the sample coordinates; arrows in the left plot show the progression of time.

In order to identify similarities between the structures of the mycobiota in the different samples analysed using NGS, we performed principal coordinate analysis (PCoA) on the weighted unifrac distances of the samples (Lozupone *et al*., 2013). In Fig. 3A, we show the results highlighting the gradual change in the samples over time. The data show that, despite the different dynamics, the Pedrotti and Pisoni musts showed a very similar dynamics, while the Poli fermentation quickly differentiated from the others. To highlight the species driving these different dynamics, on the same coordinate system we show the eight dominant genera (these genera showing the highest average relative abundance across all the samples), namely *Hanseniaspora*, *Saccharomyces*, *Botryotinia*, *Candida*, *Aureobasidium*, *Penicillium*, *Pichia* and *Starmerella*, plotted as coloured points with a size proportional to their mean relative abundance across all samples (Fig. 3B). As expected, the *Saccharomyces* genus was highly abundant in all the musts during the final phases of fermentation. However, from this data and by comparing the relative abundance of the OTUs identified in the four later samples (T14, T21, T54 and T77) (Fig. S2), we observed that starting from the second week of fermentation (T14), the Poli must was significantly enriched in *Hanseniaspora osmophila* and contained a smaller fraction of *S. cerevisiae* than the samples from the other wineries (Welch's t‐test *P* < 0.05, Fig. S3A).

To take into account the effect of the changing size of the mycobiota (Fig. 2A, for the most abundant genera see Fig. S4) on the differentiation of the samples, we performed principal component analysis (PCA) on the absolute abundance of the OTUs, calculated as the product of the relative abundance multiplied by total fungal DNA, quantified using qRT‐PCR (Fig. S5A). This analysis showed that two samples (T21 and T56) from the Pisoni fermentation clearly differed from the rest of the samples (first PCA component, 96.92% of explained variance) because of the higher amount of *S. cerevisiae* (Fig. S5B). In addition, PCA of scaled (zero mean, unit standard deviation) OTUs absolute abundance highlighted the higher abundance of *Penicillium bialowiezense*, *Nakazawaea holstii*, *Pichia membranifaciens* and *Candida zemplinina* in the Pisoni samples at T21 (first PCA component, 31.53% of explained variance; Fig. S5C).

### Core mycobiota of Vino Santo fermentation

**Figure 4:**
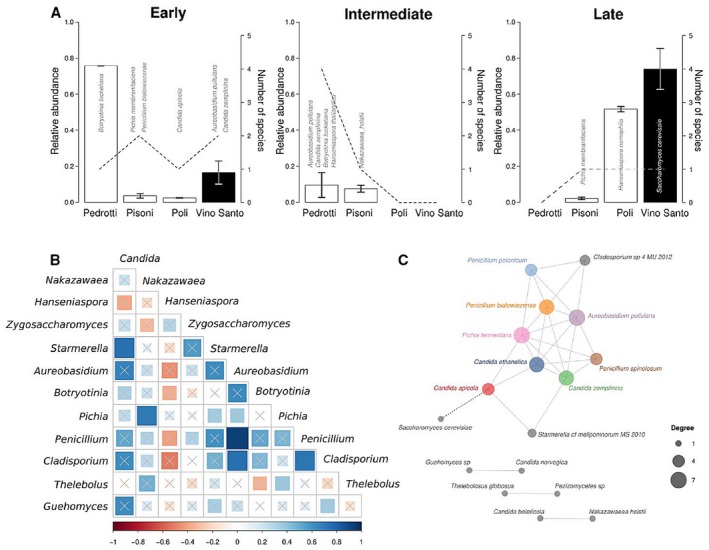
Vino Santo mycobiota. A. Average relative abundance (bars) and number of winery‐specific species (dashed lines) during the three phases of fermentation, early (0–7 days, T0‐T7), intermediate (14–21 days, T14‐T21) and late (54–77 days, T54‐T77). The fungal species present in fermentation at all the wineries make up the ‘Vino Santo core’. Filled bars indicate the average relative abundance of the Vino Santo fermentation core. The error bars indicate the standard error of the mean. The names of the species belonging to various identified groups are superimposed on (or written above) the corresponding bars; B. Absolute abundance correlation matrix of genera not having linear progression with time (Spearman *P* ≥ 0.05). Couples of correlated genera were selected when Spearman correlation resulted in r > 0.7 and *P* < 0.05. Crossed squares indicate non‐significant pairs (*P* ≥ 0.05). C. Correlation network of species selected as being correlated with each other in terms of relative abundance and having a linear progression with time. Pairs of correlated species were selected when Spearman correlation resulted in r > 0.7 and *P* < 0.05. Significantly correlated pairs of species were then selected as significantly correlated with time (linear regression of both species having *P* < 0.05).

The results shown so far indicate that the Vino Santo fermentation is a dynamic process with considerable variations in the size, structure and composition of the mycobiota (see also Fig. S2). It is therefore not surprising that we failed to identify fungal species consistently present in all fermentations at all times (Table S4). Even considering each fermentation separately only in the Pisoni must we could find one species, *P. membranifaciens*, that was present in every stage of fermentation with a relative abundance of at least 1% (Table S4). To understand whether it was possible to break down the dynamics of fermentation into distinct phases and to assess whether there was a core mycobiota for these phases, we performed hierarchical clustering of the relative abundances of the Poli and Pisoni samples separately (Fig. S2). We found that in both these fermentation processes three phases could be distinguished: an early phase (T0 and T7), an intermediate phase (T14 and T21) and a late phase (T56 and T77; Fig. S2). For each of these phases separately, we tested whether it was possible to identify fungal species shared by all the wineries. In the early phase of fermentation (T0 and T7), we found two fungal species, namely *A. pullulan*s an*d C. zemplinin*a that were present in each sample, representing 27.7 ± 11.1% and 5.3 ± 0.8% (average ± standard error of OTU relative abundances in samples) of the fungal populations respectively (Fig. 4A and Table S4). No common species were found in the three wineries during the intermediate phases of fermentation (T14–T21; Fig. S5A) probably because of the large change in population size during this phase (Fig. 2A). During the final stages of fermentation, *S. cerevisiae*, the fungal species known to be the main driver of alcoholic fermentation, was present with high abundance in all the wineries, representing on average 99.6%, 88.1 ± 10%, and 47.1 ± 2.8 % in the Pedrotti, Pisoni and Poli fermentations respectively. *Hanseniaspora osmophila* was highly represented in the fermentation of the Poli winery (Fig. 4A and Fig. S3A), explaining the lower abundance of *S. cerevisiae* in late fermentation phases. Although present with different relative abundance in the three must fermentations, *S. cerevisiae* showed a consistent dynamic in the three wineries (Fig. S3B). In contrast with other fungal species, in all the wineries the relative abundance of *S. cerevisiae* gradually increased over time, reaching a relative abundance close to 100% for some fermentations (Welch's *t*‐test *P* < 0.05; Fig. S3B). In contrast, the relative abundance of the environmental species *Starmerella meliponinorum*, *Candida apicola*, *P. bialowiezense* and *Alternaria alternata* already underwent a drastic decrease after T14 in all the wineries, suggesting their inability to survive, either due to the stressful environment of the fermenting must or due to competition with other fitter species (Fig. S3B). The relative abundance of *C. zemplinina*, *Candida ethanolica* and *A. pullulans*, environmental species more resistant to the fermenting must environment, saw a more limited decrease until they almost disappeared after T56 (Welch's t‐test *P* < 0.05, Fig. 4A and Fig. S3B).

To further characterize the collective dynamics of fungal species in Vino Santo fermentation, we calculated Spearman's correlations for each pair of fungal OTUs with a relative abundance exceeding 1%. Significant correlations were found between *Candida* and *Starmerella* genera, *Nakazawaea* and *Pichia* genera and *Aureobasidium*, *Penicillium* and *Cladosporium* genera (Fig. 4B). Of these, the latter group of correlated genera was the only one for which relative abundance decreased during the fermentation progression (Fig. S6A). *Starmerella* and *Candida* spp., initially present in small proportions in fresh must, underwent a temporary increase after the first week of fermentation (Fig. S6B). The *Candida* genus present in Vino Santo fermentation was mainly made up of *C. zemplinina*, *C. apicola* and *C. ethanolica* (Fig. S7). All these three species are osmotolerant, while only *C. ethanolica* is able to survive at up to 14% ethanol concentrations (25).

We then explored the existence of correlations between the absolute abundances of each pair of fungal species. Through Spearman correlation analysis, *S. meliponinorum* was found to be positively correlated with *C. apicola* (r = 0.84, *P*‐value = 8.83e‐05) and *C. zemplinina* (r = 0.71, *P*‐value = 0.03) (Fig. 4C). In turn, *C. apicola* was shown to be negatively correlated with *S. cerevisiae* (r=−0.75, *P*‐value = 0.014), known to prevail at late stages of must fermentation (Polsinelli *et al*., 1996).

### Bacterial component of Vino Santo fermentation

Alongside the prominent role of the fungal component of the microbiota in alcoholic fermentation, it has been shown that bacteria can contribute to the organoleptic characteristics of wine (Swiegers *et al*., 2005), despite the general sensitivity of bacterial species to the presence of alcohol. To explore the potential contribution of the bacterial population to Vino Santo fermentation, we determined the size of the microbiota's bacterial component using qRT‐PCR DNA quantification, with universal primers specific for the V1–V3 hyper variable region of the 16 rDNA gene in the Poli samples. In the same region and for the same samples, we further characterized the structure of the bacterial population using 454‐pyrosequencing (results are described in further detail in the *Supplementary results*). The total size of the bacterial component increased during the first week of fermentation, followed by a rapid decrease that continued at a lower rate on subsequent fermentation days (Fig. S8A), probably due to the progression of alcoholic fermentation, which made the environment unfavourable for the survival of bacterial species sensitive to high levels of ethanol. As a consequence, the bacterial populations showed reduced diversity over time (described either by the Chao1 and Shannon diversity indexes, or by the number of observed OTUs; Fig. S9). The taxonomic structure of the bacterial component of the microbiota also changed in a time‐dependent manner. The relative abundance of lactic acid bacteria, mainly belonging to the *Oenococcus* genus, increased over the fermentation process until it reached a maximum (30% of the bacterial population) at T21 (Fig. S10).

Given that bacteria and fungi can influence each other through either metabolic competition/synergy or modifications to the environment (Wargo and Hogan, 2006), we evaluated the existence of correlations between the absolute abundance of fungal species and bacterial genera. Strong correlations (Spearman's r = 1, *P* < 0.001) were found between the yeasts species *C. ethanolica* and *Pichia fermentans* and the bacteria genera *Hymenobacter*, *Psychrobacterium* and *Ralstonia* (Fig. S8c). *Candida ethanolica* and *P. fermentans* are known to persist in must during alcoholic fermentation and to produce metabolites contributing to the organoleptic characteristics of the final product (Moreno‐Arribas and Polo, 2008).

### Microbial populations and the chemical–physical characteristics of fermentation

**Figure 5:**
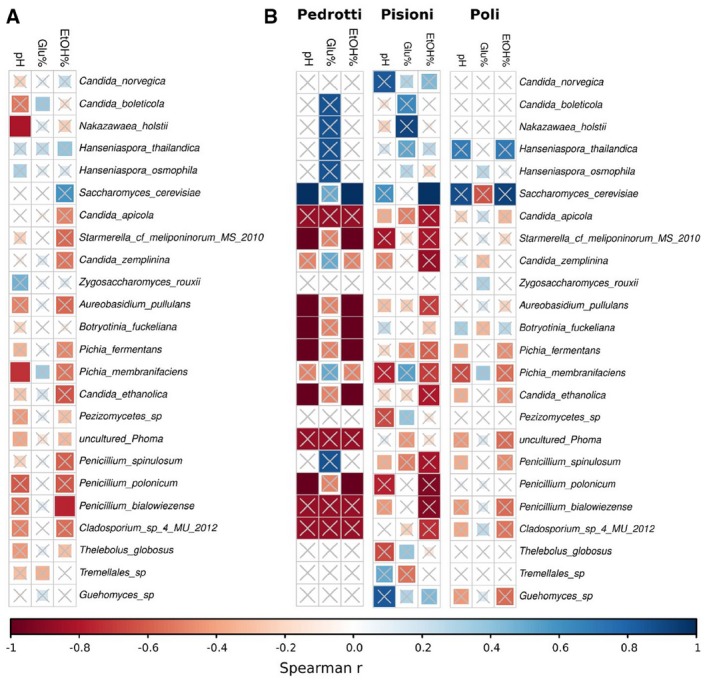
Spearman's correlations with fungal OTU relative abundances and chemical factors. A. computed using the entire dataset. B. computed using data divided by winery. Crossed squares indicate non‐significant pairs (*P* ≥ 0.05).

The results presented so far indicate that the size and structure of microbiota change over time during the fermentation process. Given the known sensitivity of many microbial species to environmental conditions, we searched for correlations between the absolute abundance of both bacteria and fungi and three environmental factors known to shape the progress of fermentation, namely (i) glucose concentration, (ii) ethanol concentration and (iii) pH. Until T21, while ethanol production was almost absent, the concentration of glucose underwent a temporary increase (Fig. S11A–B). The situation changed (with ethanol concentration starting to increase and glucose concentration starting to decrease) at T21, the time at which *S. cerevisiae* relative abundance started to increase (Fig. S3B). The pH values of must correlated with ethanol concentration (Spearman's r = 0.576, *P* = 0.025) and showed a negative correlation with *P. membranifaciens* relative abundance (Spearman's r = −0.721, *P* = 0.002). The increase in ethanol concentration is considered to be the principal factor selecting microbial populations during fermentation, and is thus supposed to be negatively correlated with the abundance of sensitive species and positively correlated with that of resistant species. Nevertheless, while the total amount of fungi (evaluated through qRT‐PCR on the ITS1 region) was shown to be correlated to ethanol concentration (Spearman's r = 0.571, *P* = 0.026; Fig. S11C), only the relative abundance of *P. bialowiezense* was negatively correlated with ethanol concentration (Spearman's r=−0.762, *P* = 0.003, Fig. 5A). Furthermore, none of the relative abundances of the identified fungal taxa (at genus, family or order level) were correlated with glucose concentration, when considering all the wineries. Similarly, no bacterial taxa (whether at genus, family or order level) correlated with either ethanol or glucose concentrations (data not shown). The lack of correlations between the relative abundance of fungal species and single chemical factors can be explained by two considerations: (i) the survival of microbes in the stressful must environment is not shaped by a single factor, but more probably by a combination of several different factors, (ii) the three wineries have microbial and metabolomic dynamics that are so different that shared patterns cannot be identified. To explore the first possibility, we tested the existence of linear relations between OTU absolute abundances and combinations of chemical factors through stepwise regression analysis (linear regression analysis using the function stepAIC of the MASS r package). The absolute amount of *Wickerhamomyces anomalus*, *P. bialowiezense, Guehomyces*_sp, *Cladosporium*_sp_4_MU_2012, *Torulaspora delbrueckii* and *Nakazawaea holstii* showed negative linear correlation with ethanol concentrations and with the combination of all the evaluated chemical parameters, and positive linear correlation with the combination of ethanol and either glucose concentrations or pH (stepwise regression analysis, bidirectional elimination, *P* < 0.05, details in Table S5), indicating their preference for an environment characterized by a low ethanol concentration, high glucose concentration and mild acidic pH, as is usually the case for must in the early stages of fermentation. *Candida norvegica and C. zemplinina* instead had positive linear correlation with the combination of ethanol and glucose concentration, and negative correlation with the combination of all the chemicals. *Candida ethanolica* and *A. pullulans* showed negative linear correlation with ethanol and the combination of all chemical parameters and positive linear correlation with the combination of ethanol and glucose (stepwise regression analysis, bidirectional elimination, *P* < 0.05, Table S5).

A factor hampering the identification of correlations between fungal OTUs and physical–chemical characteristics was the significant variability of some of the chemical parameters in the different wineries. In particular, the Pedrotti must samples were significantly more acidic (low pH) than the must from the other two wineries (Wilcox signed‐rank test *P* < 0.05; Table S6). In addition, the Pisoni fermentation showed a total acidity higher than the Poli fermentation (Wilcox signed‐rank test *P* = 0.029; Table S6). We thus investigated whether these differences had an effect on microbiota, through Spearman correlation analysis of fungal OTU relative abundances and the chemical values of the three wineries separately (Fig. 5B). We found negative correlation between pH and ethanol concentration and several species (*S. meliponinorum*, *A. pullulans*, *Botryotinia fuckeliana*, *P. fermentans*, *C. ethanolica* and *Penicillium polonicum*) in the Pedrotti must, which being more acidic, represented the most stressful environment. These species, likely to be of environmental origin probably survived during fermentation until the conditions became prohibitive.

## Discussion

Sweet wines are produced by a particular type of fermentation, the secrets of which are handed down through generations of winemakers. These sweet wines are a particularly interesting model for studying the dynamic process of grape must transformation into wine, since the freshly crushed must presents one of the richest and most complex microbial communities, which functions as inoculum in spontaneous fermentations. This initial mycobial diversity slowly evolves in extremely stressful conditions, dominated by high sugar and low initial temperatures, and at the end of the process, only a few strains survive. In this work, we investigated in detail the microbial dynamics of typical, traditional sweet wine production in the Trentino region, through parallel characterisation of microbiota mycobiota and fermentation dynamics and metabolite profiles. Using absolute quantification of microbial DNA by qRT‐PCR in combination with high throughput sequencing of ITS 1‐4, we found that the fungal component of the microbiota, initially constituted by a high number of different species, grew in size but became more simplified after the first 2 weeks of fermentation and was ultimately dominated by a small number of species, probably either favoured by the particular biochemical conditions or able to out‐compete other species. Although it has been shown that the DNA of dead cells can persist for a long time in different matrices (Josephson *et al*., 1993; Nocker and Camper, 2006), our findings on the growing size of the fungal component of the mycobiota obtained using qRT‐PCR suggest that the potential bias due to persistence of the DNA of dead cells does not hamper our ability to detect significant differences, and that our approach makes it possible to balance this effect and permits determination of the mycobiota species dynamically changing in a significant way during fermentation. In addition, the observed decrease in the absolute amount of several species (i.e. *P. membranifaciens*), which became almost undetectable after an initial increase, suggested that in our case, the DNA of dead species did not accumulate in the fermenting must, thus confirming the robustness of our observations.

The initial microbiota of the three wineries, encompassing both fungal and bacterial environmental species and genera (*A. pullulans*, *S. meliponinorum, Penicillium*, *Candida, Sphingomonas*, *Propionibacter* and *Corynebacterium*; (Teixeira *et al*., 2003; Leveau and Tech, 2011; Milanović *et al*., 2013; Campisano *et al*., 2014; Pinto *et al*., 2014), was different at the three sites. This could be ascribed either to stochastic changes on the microbiota, unrelated to the different grape origins, or to differences in the ecological niche even in the same geographical region. The possibility for vineyards in the same geographical region to host extremely diverse microenvironments, resulting in significantly different microbiota composition, has been recently proven (Bokulich *et al*., 2014). Whatever is the condition that shaped the initial microbial populations, the structure of the mycobiota became quickly uncorrelated with its initial state, as shown by correlation analysis during the course of the fermentation, indicating evolution through rapid selection associated with wine fermentation. The identification of correlations between combinations of physical–chemical factors and fungal abundance indicates a role for self‐produced stressors in shaping the microbiota. Natural selection during must fermentation seems to be stronger than the effects of which species make it into particular musts. This indication extends to human‐related environments the observation already made in wild environments, namely that microbial populations are influenced more by micro‐evolution in their ecological niche than by their geographical location (Morrison‐Whittle and Goddard, 2015). The first global analysis of the dynamics of dried grape wine fermentation in a well defined and geographically limited area shows how the early stages of the process in slow fermentation are characterized by a high level of biodiversity. As observed in other fermentations, glucose and ethanol concentrations and must pH have a significant role in shaping the microbial population, with must acidity playing the predominant role, both in selecting the initial fungal population (Charoenchai *et al*., 1998) and in defining the fermentation properties of fungi (Liu *et al*., 2015). Although starting from very different and complex situations, the three mycobiota converged to a common pattern, mainly made up of *S. cerevisia*e, while the species dynamics differed significantly in at least in one of the three wineries. The harvesting time, the duration of the drying period, the eventual clarification of must and in general the maintenance of the fermenting must at low temperatures are all factors that can radically influence the progression of the fermentation, having an impact on both the physical–chemical characteristics of the must (i.e. its osmolarity) and on microbial populations. Slight modification of the protocol, such as the clarification, by cooling step performed only in the Poli winery before the beginning of fermentation, could be one of the causes for the deviation from the common results in the final phases of the process, when the fermentation at the Poli winery was surprisingly carried out by the concerted action of *S. cerevisiae* and *H. osmophila*, a cryotolerant species previously identified in sweet wine production (Granchi *et al*., 2002). Thus, slight modifications in the type of vinification procedure adopted could have an effect on the speed with which *S. cerevisiae*, initially rare in fresh must, takes over fermentation. The combination of pH and ethanol, together with the concerted action of competing species could explain the rapid disappearance of *B. fuckeliana*, the ‘noble rot’ dominating grapes during the drying process and found in all Vino Santo fresh musts only at the beginning of fermentation (Azzolini *et al*., 2013). Our global results reveal the outcome of the biological warfare unleashed by *S. cerevisiae*, taking advantage of the high level of glucose present in fresh must to aerobically ferment and produce ethanol (Crabtree effect), representing a further stress for sensitive microbes, and then respiring ethanol to acetic acid to produce a lethal weapon that allows only *Saccharomyces* to survive, in agreement with the make–accumulate hypothesis (Goddard, 2008). The particular nature of this slow fermentation and low temperature, probably explains the surprising finding in this study that another species, *H. osmophila*, can survive together with *S. cerevisiae* in some fermentation of Vino Santo.

## Experimental procedures

### Samples collection

Must samples were collected in three wineries – Poli (Santa Massenza, Trento, Italy), Pedrotti (Pietramurata, Trento, Italy) and Pisoni (Pergolese, Trento, Italy) – located in the Valle dei Laghi, a valley located in the Italian Alps near Trento (Italy). None of the producers studied has inoculated Vino Santo fermentations with selected yeast strains for the past 5 years, and during this period all of them were certified organic wine growers, using limited amounts of sulfides and eliminating the use of antifungal drugs and systemic treatments. Must samples were collected once a week from the start to the end of fermentation (corresponding to complete consumption of must sugar). Fermentation was carried out in 1000 litre tanks and at least 50 ml of must were collected after stirring the tank content at each sampling. The fermentation lasted 3 months, and 12 samples were collected from each winery. Samples were collected in sterile tubes then stored at –80°C until DNA extraction. A summary of the collected samples is shown in Table S1).

### DNA extraction

Must samples were stored at −80°C until DNA extraction. Extraction of DNA was carried out each time from 2 ml thawed must. Must was centrifuged 30 min at 14 000 g and at 4°C, and the pellet was dissolved in 2 ml TE buffer. Must was centrifuged again for 15 min at 14 000 g at 4°C, and the pellet was dissolved in 300 μl TE buffer. Extraction of DNA was then carried out with the FastDNA Spin Kit for Soil (MP biomedicals) following the manufacturer's instructions. When the DNA extraction resulted in low yield (less than 10 ng/μl) or poor quality (260/230 ratio lower than 1.9), DNA extraction was repeated on new sample aliquots by adding small amounts of polivinilpirrolidone and vortexing the must for 15 s, then the must was incubated at 60°C for 1 h to eliminate tannins and polyphenols, and from 1.1 μl to 100 μl ß‐mercaptoethanol were added to unfold proteins before the use of the FastDNA Spin Kit for soil.

### Microbiota profiling and sample selection for meta‐taxonomic analysis

To select samples to be further analysed using high throughput 454‐pyrosequencing techniques, we profiled all samples using PCR‐RFLP on extracted fungal DNA. Fungal populations were analysed by amplifying the ITS1‐5.8S‐ITS2 regions (primer sequences in Table S2) and digesting the amplified DNA with *HaeIII* and *HinfI* restriction enzymes as previously described (Dlauchy *et al*., 1999). Patterns of RFLP were generated with the GelAnalyzer2010a software (freeware) assigning a bp length to the fragments obtained after digestion and applying 5% tolerance (bands were considered identical only if their size deviated by less than 5% of the average size of the group of bands they were assigned to). Band profiles were then converted into absence/presence profiles and, to compare the converted PCR‐RFLP profiles, a dendrogram was constructed as previously described (De Filippo *et al*., 2010) by maximum parsimony using the phylip program (Felsenstein, 1989). Samples were selected as representative of the widest fungal population variation and covering as much of the fermentation period as possible.

### Bacterial and fungal quantification using quantitative RT‐PCR

To monitor the dynamics of fermentation as a function of time, we measured the total amount of bacterial and fungal DNA in the samples. Total bacterial and fungal DNA were quantified by using universal primers specific for either the V1–V3 region of 16S rRNA gene (Baker *et al*., 2003) for bacteria, or the ITS1 region (Findley *et al*., 2013) for fungi (primer sequences are listed in Table S2). The primers used to quantify fungal DNA differed from those used for pyrosequencing to ensure the homogeneity of the length of the amplified DNA fragment, which ranges from 400 bp to 880 bp if ITS1 and ITS4 primers are used.

Quantitative RT‐PCR was carried out following the protocol recently described (Bittinger *et al*., 2014) with minor changes. Standard curves were constructed using PCR products of the 16S rRNA gene or of the ITS1 region. The PCR product was purified from agarose gel and quantified with Quant‐iT PicoGreen dsDNA Assay Kit (Life Technologies). Serial dilution was performed and 10 ng, 7.5 ng, 5 ng, 2.5 ng, 1 ng, 0.5 ng, 0.05 ng and 0.005 ng of PCR products were used for calibration. Real‐time PCR was performed with the Light Cycler(R) 480 (Roche) using optical grade 96‐well plates. The PCR reaction was performed in a total volume of 12.5 μl using the KAPA SYBR(R) Fast qPCR Kit (KAPABiosystems). Each reaction included 6.25 μl KAPA SYBR FAST qPCR Master Mix (2X), 0.25 μl of each primer (10 μM) and 1 ng template DNA. The reaction conditions for amplification of DNA were 40 thermal cycles at 95°C for 15 s, 60°C or 58°C (bacterial and fungal DNA, respectively) for 30s and 72°C for 10s. To determine the specificity of amplification, analysis of the product melting curve was performed after the last cycle of each amplification. Finally, absolute fungal and bacterial abundances were calculated as the product of OTU relative abundance and the respective total fungal or bacterial DNA amount detected in the sample.

### PCR amplification of the V1–V3 region of bacterial 16S rRNA genes and of the ITS1‐5.8S–ITS2 fungal DNA region

The 16S rRNA genes were amplified by using special fusion primer sets specific for V1–V3 hyper‐variable regions (Table S2). The ITS1‐5.8S–ITS2 region was amplified by using a fusion primer set specific for ITS1‐5.8S–ITS2 regions (Table S2).

The forward primer sequences were made up of: the ‘LIB‐L’ primer A sequence specific for ‘Lib‐L’ chemistry and ‘One‐Way Reads’ sequencing methods (Roche, Branford, CT), the key sequence TCAG, the bar code (Multiple IDentifier) sequence (specific for any sample) and the forward primer sequence. The reverse primer contained the ‘Lib‐L’ primer B sequence, the key sequence TCAG and the reverse primer sequence. For each sample, a PCR mix of 25 μl was prepared containing 1X PCR buffer, 1.25 U of FastStart High Fidelitypolymerase blend (Roche) and dNTPs from the FastStart High Fidelity PCR system (Roche), 0.4 μM of each primer (PRIMM, Milano) and 10 ng of gDNA. Thermal cycling consisted of initial denaturation at 94°C for 3 min followed by 25 cycles (35 for fungal ITS) of denaturation at 94°C for 15 s, annealing at 60°C (58°C for fungal ITS) for 45 s, and extension at 72°C for 1 min, with a final extension of 8 min at 72°C.

### Library construction and pyrosequencing

Products of PCR were analysed using gel electrophoresis and cleaned using the AMPure XP beads kit (Beckman Coulter, Brea, CA, USA) following the manufacturer's instructions. Products of the different samples were quantified via quantitative PCR using the Library quantification kit – Roche 454 titanium (KAPA Biosystems, Boston, MA) and pooled in an equimolar way in a final amplicon library. Carried out was 454 pyrosequencing on the GS FLX + system using XL + chemistry, following the manufacturer's recommendations.

### Pyrosequencing quality control

Pyrosequencing produced a total of 262 783 reads of 16S rDNA (for eight sequenced samples) and 235 554 reads of ITS (for 16 sequenced samples). The sequences were assigned to samples according to sample‐specific barcodes. This allowed us to collect FASTA formatted files containing an average of 18 637.17 ± 8815.27 (SD) V1‐V3 16S rRNA sequences and 14 809.88 ± 14 681.50 (SD) ITS1‐5.8S‐ITS2 region sequences per sample. Sequences were then checked for the following criteria: (i) almost perfect match with bar code and primers, (ii) length of at least 150 nucleotides (bar codes and primers excluded) and (iii) no more than two undetermined bases (denoted by N). By ‘almost perfect match’ we mean that one mismatch/deletion/insertion is allowed in the bar code, idem for the primer. Data were submitted to the European Nucleotide Archive with accession number PRJEB7999 (http://www.ebi.ac.uk/ena/data/view/PRJEB7999). The correspondence between submitted data IDs and samples is shown in the supplementary file ‘sample_data.txt’.

### Data analysis

Raw data files generated by the Roche 454 sequencer were de‐multiplexed using Roche's sfffile software. Reads were pre‐processed using the micca pipeline (Albanese *et al*., 2015). Forward and reverse primers trimming and quality filtering were performed using micca‐preproc (parameters ‐f AGAGTTTGATCMTGGCTCAG ‐r TTACCGCGGCTGCTGGCAC ‐O 16 ‐l 300 ‐q 25 for 16S data and ‐f GTTTCCGTAGGTGAACCTGC ‐r TCCTCCGCTTATTGATATGC ‐O 16 ‐l 400 ‐q 20 for ITS data), truncating reads shorter than 300 (16S) and 400 (ITS) bp respectively. *De novo* sequence clustering, chimera filtering and taxonomy assignment were performed using micca‐otu‐denovo (parameters –s 0.97 –c): OTUs were assigned by clustering the sequences with a threshold of 97% pairwise identity, and their representative sequences were classified using rdp software version 2.7 (Wang *et al*., 2007) on the 16S data and using blast against the unite database (Kõljalg *et al*., 2013) (release 09/02/2014) on the ITS data. For ITS data, multiple sequence alignment (MSA) and phylogenetic tree inference were performed using the online version of T‐Coffe (Notredame *et al*., 2000). The taxonomies of all the representative sequences identified in the samples were further checked by comparing the blast results of the taxonomic classification against the rdp classifier (v. 2.8) and by manually blasting each sequence through the National Center for Biotechnology Information nucleotide collection database (http://blast.ncbi.nlm.nih.gov/Blast.cgi?PROGRAM=blastn&PAGE_TYPE=Blast Search&LINK_LOC = blasthome). The results of this comparison are shown in Table S3. The taxonomic assignment of the vast majority (77.19%) of sequences was confirmed by all the approaches; these sequences which were not univocally assigned to the same taxon at the species level, still matched at the genus level. To ensure reproducibility and comparability of the results, we did not modify the automatic blast taxonomic assignment, in agreement with the accepted standards in the field. Sampling heterogeneity was reduced by rarefaction (3500 sequences per sample for 16S data and 3,119 for ITS data). Alpha (within‐sample richness) and beta‐diversity (between‐sample dissimilarity) estimates were computed using the phyloseq r package (McMurdie and Holmes, 2013). Two‐sided, unpaired Welch *t*‐statistics were computed using the function mt() in the phyloseq library (McMurdie and Holmes, 2013), and the *P*‐values were adjusted for multiple comparison controlling the family‐wise Type I error rate (minP procedure) (Westfall and Young, 1993). Correlations among fungal species, bacterial genus and chemicals were evaluated with the psych r package (Revelle, 2014). False discovery rate (FDR)‐adjusted *P*‐values were computed using the Benjamini–Hochberg procedure (Benjamini and Hochberg, 1995).

Stepwise regression analysis was carried out by applying the stepAIC function of the mass r package (Venables and Ripley, 2002). Briefly, regression was carried out on dependent variables (OTU relative abundances) and different combinations of explanatory variables (chemical indexes alone or combinations of variables). Models were classified on the basis of their Akaike information criterion, and the combination of variables producing the best model was selected for each taxonomic unit.

## Conflict of Interest

None declared.

## Supporting information

Supplementary Results: Analysis of the bacterial population of Poli vineyard fermentation.
**Fig. S1.** Fungal biodiversity in must samples.
**Fig. S2.** Compositional profiles of Vino Santo fermentation mycobiota.
**Fig. S3.** Details of fungal species relative abundance with comparison of wineries and fermentation times.
**Fig. S4.** Relative abundances of the six most abundant fungal genera.
**Fig. S5.** principal component analysis of fungal ITS absolute abundance.
**Fig. S6.** Time progression of genera found to be correlated to each other.
**Fig. S7.** Composition of the *Candida* genus as represented in Vino Santo must during the fermentation process.
**Fig. S8.** Bacteria population during fermentation.
**Fig. S9.** Bacterial population alpha diversity estimated according to three different indexes.
**Fig. S10.** Relative abundance of lactic acid bacteria) over the fermentation period.
**Fig. S11.** Ethanol and glucose concentrations during the fermentation of different Vino Santo musts.
**Table S1.** Summary of samples and analysis. ‘x’ indicates that the analysis was carried out on the sample. For details on the experimental procedures please refer to the ‘*material and methods*’ section.
**Table S2.** Sequences of primers used in this study for various purposes.
**Table S3.** Comparison of taxonomic assignments. (see .xls file).
**Table S4.** Fungal species characterising either Vino Santo fermentation or fermentation at the three wineries.
**Table S5.** Summary of the results of stepwise regression analysis carried out on OTU relative abundance (observations) and the principal chemicals measured (variables). False discovery rate adjusted *P*‐values were computed using the Benjamini–Hochberg procedure (Benjamini and Hochberg, 1995) (see .xls file).
**Table S6.** Results of Wilcox signed‐rank tests to compare chemical values of must from the three wineries. The columns designed as ‘P’ indicate the *P*‐values resulting from the Wilcox test after FDR correction (FDR adjusted *P*‐values were computed using the Benjamini‐Hochberg procedure (Benjamini and Hochberg, 1995).
Sample data: Correspondence between submitted data IDs and samples. Data have been submitted to the European Nucleotide Archive with the accession number PRJEB7999 (http://www.ebi.ac.uk/ena/data/view/PRJEB7999). (.txt file).
