# Supplementary material for: Dynamic changes in microbiota and mycobiota during spontaneous ‘Vino Santo Trentino’ fermentation
Source: Microb Biotechnol. 2016 Jan 18;9(2):195–208. doi: 10.1111/1751-7915.12337 (PMC4767281; doi:10.1111/1751-7915.12337)
Supplement: Supplementary file 1 — Supplementary Results: Analysis of the bacterial population of Poli vineyard fermentation. Fig. S1. Fungal biodiversity in must samples. Fig. S2. Compositional profiles of Vino Santo fermentation mycobiota. Fig. S3. Details of fungal species relative abundance with comparison of wineries and fermentation times. Fig. S4. Relative abundances of the six most abundant fungal genera. Fig. S5. principal component analysis of fungal ITS absolute abundance. Fig. S6. Time progression of genera found to be correlated to each other. Fig. S7. Composition of the Candida genus as represented in Vino Santo must during the fermentation process. Fig. S8. Bacteria population during fermentation. Fig. S9. Bacterial population alpha diversity estimated according to three different indexes. Fig. S10. Relative abundance of lactic acid bacteria) over the fermentation period. Fig. S11. Ethanol and glucose concentrations during the fermentation of different Vino Santo musts. Table S1. Summary of samples and analysis. ‘x’ indicates that the analysis was carried out on the sample. For details on the experimental procedures please refer to the ‘material and methods’ section. Table S2. Sequences of primers used in this study for various purposes. Table S3. Comparison of taxonomic assignments. (see .xls file). Table S4. Fungal species characterising either Vino Santo fermentation or fermentation at the three wineries. Table S5. Summary of the results of stepwise regression analysis carried out on OTU relative abundance (observations) and the principal chemicals measured (variables). False discovery rate adjusted P‐values were computed using the Benjamini–Hochberg procedure (Benjamini and Hochberg, 1995) (see .xls file). Table S6. Results of Wilcox signed‐rank tests to compare chemical values of must from the three wineries. The columns designed as ‘P’ indicate the P‐values resulting from the Wilcox test after FDR correction (FDR adjusted P‐values were computed using the Benjamini‐Hochberg proced [file MBT2-9-195-s001.zip › MBT2_12337_supp-0002-Stefanini_Supplementary_re-submitted_version.pdf]

Supplementary material
for

Dynamic changes in microbiota and mycobiota during spontaneous "Vino Santo Trentino" fermentation

Supplementary Material content:
Supplementary Results: Analysis of the bacterial population of Poli vineyard fermentation

Supplementary Figures:
Figure S1: Fungal biodiversity in must samples.
Figure S2: Compositional profiles of Vino Santo fermentation mycobiota.
Figure S3: Details of fungal species relative abundance with comparison of wineries and fermentation times.
Figure S4: Relative abundances of the six most abundant fungal genera.
Figure S5: PCA of fungal ITS absolute abundance.
Figure S6: Time progression of genera found to be correlated to each other.
Figure S7: Composition of the *Candida* genus as represented in Vino Santo must during the fermentation process.
Figure S8: Bacteria population during fermentation.
Figure S9: Bacterial population alpha diversity estimated according to 3 different indexes.
Figure S10: Relative abundance of LAB (Lactic Acid Bacteria) over the fermentation period.
Figure S11: Ethanol and glucose concentrations during the fermentation of different Vino Santo musts.

Supplementary Tables (captions):
Table S1: Summary of samples and analysis. “x” indicates that the analysis was carried out on the sample. For details on the experimental procedures please refer to the “material and methods” section.
Table S2: Sequences of primers used in this study for various purposes.
Table S3: Comparison of taxonomic assignments. (see .xls file)
Table S4: Fungal species characterising either Vino Santo fermentation or fermentation at the three wineries.
Table S5: Summary of the results of stepwise regression analysis carried out on OTU relative abundance (observations) and the principal chemicals measured (variables). FDR adjusted p-values were computed using the Benjamini–Hochberg procedure (Benjamini Y. & Hochberg Y. 1995)(see .xls file)
Table S6: Results of Wilcox signed-rank tests to compare chemical values of must from the three wineries. The columns designed as “P” indicate the p-values resulting from the Wilcox test after FDR (false discovery rate) correction (FDR adjusted p-values were computed using the Benjamini–Hochberg procedure (Benjamini Y. & Hochberg Y. 1995).
Sample data: Correspondence between submitted data IDs and samples. Data have been submitted to the European Nucleotide Archive with the accession number PRJEB7999 (http://www.ebi.ac.uk/ena/data/view/PRJEB7999). (.txt file)

<u>Figure 1:</u> Fungal biodiversity in must samples. a- alpha diversity; **b-** beta diversity estimated on Bray-Curtis distances; **b-** beta- diversity estimated on Unweighted UniFrac distances; in panels **b** and **c** the left graphs show the first two coordinates of the PCoA, carried out using the samples as cases and fungal OTUs’ relative abundance as variables; the right graph shows the same two PCoA coordinates with the most abundant fungal OTUs superimposed as coloured points, with the size being proportional to the mean relative abundance of the taxon across all samples. Grey dots in the right plots of panels **b** and **c** indicate sample coordinates.

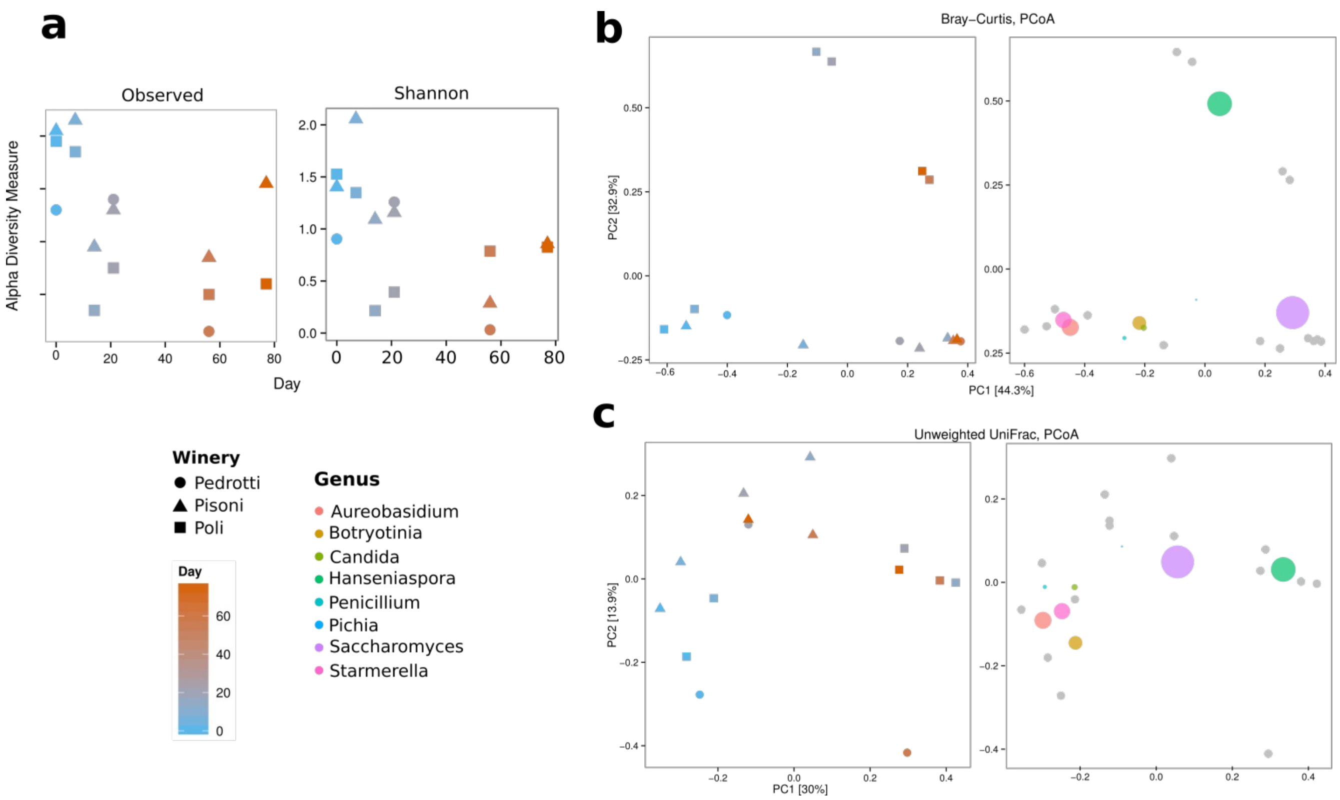

<u>Supplementary Figure 2</u>: Composition profiles of Vino Santo fermentation mycobiota. Relative abundance of operational taxonomic units (OTUs) from fungal species in Vino Santo fermentation samples. Colours correspond to species (the most abundant species are listed in the figure legend). Samples are named as shown in Fig. 1a (at the bottom of the coloured columns showing the relative abundance of the fungal species). Hierarchical clustering based on Weighted Unifrac distances between mycobiota at the time points is shown at the top of the coloured columns.

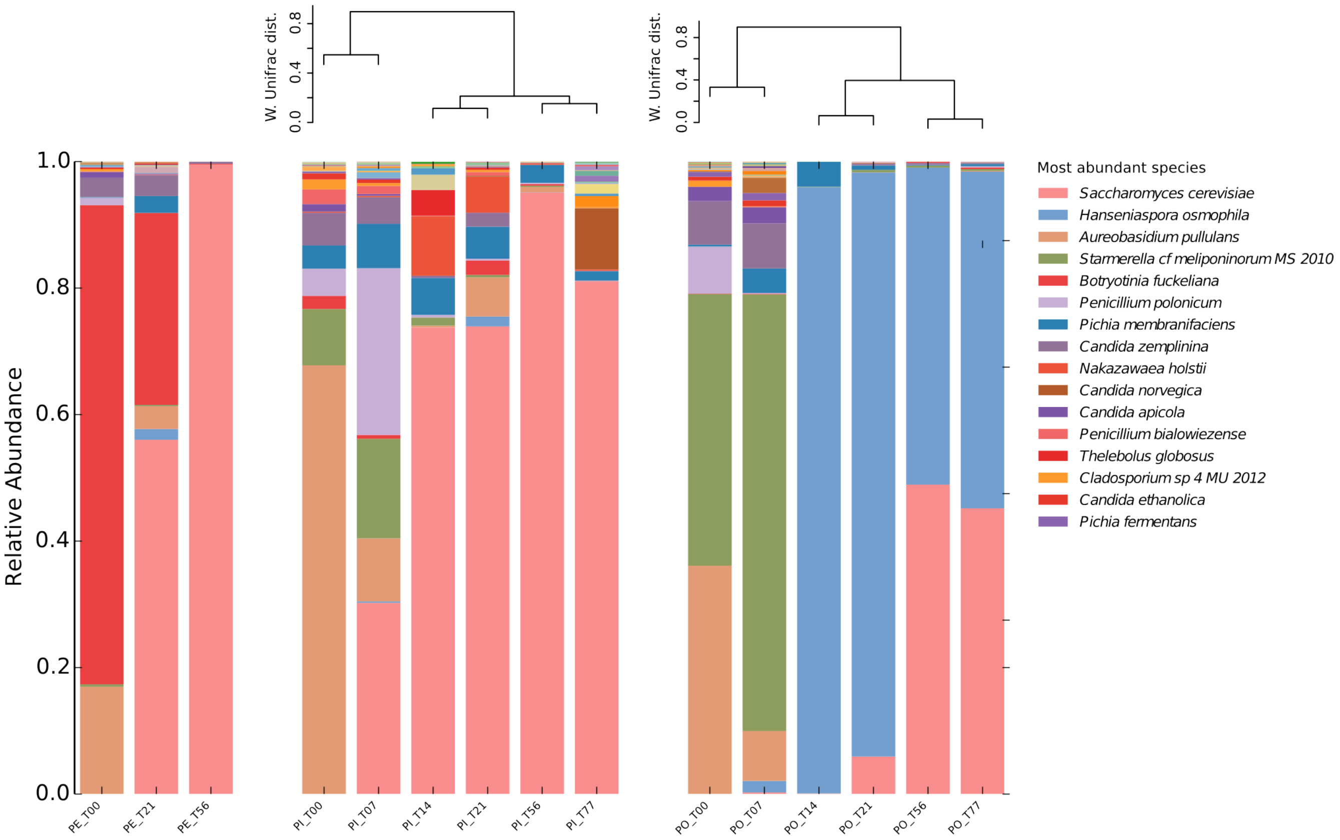

<u>Supplementary Figure 3</u>: **Details of fungal species relative abundance, with comparison between wineries and fermentation times. a-** Fungal species differentially represented at the late fermentation stage (T56-T77) of the Polo winery in comparison to the other wineries. **b-** Fungal species differentially represented for early (from 0 to 7 days' fermentation), intermediate (from 14 to 21 days' fermentation) and late fermentation (from 56 to 77 days' fermentation). t-test was carried out to compare relative abundance in different fermentation phases and only species with fdr corrected P<0.05 were selected.

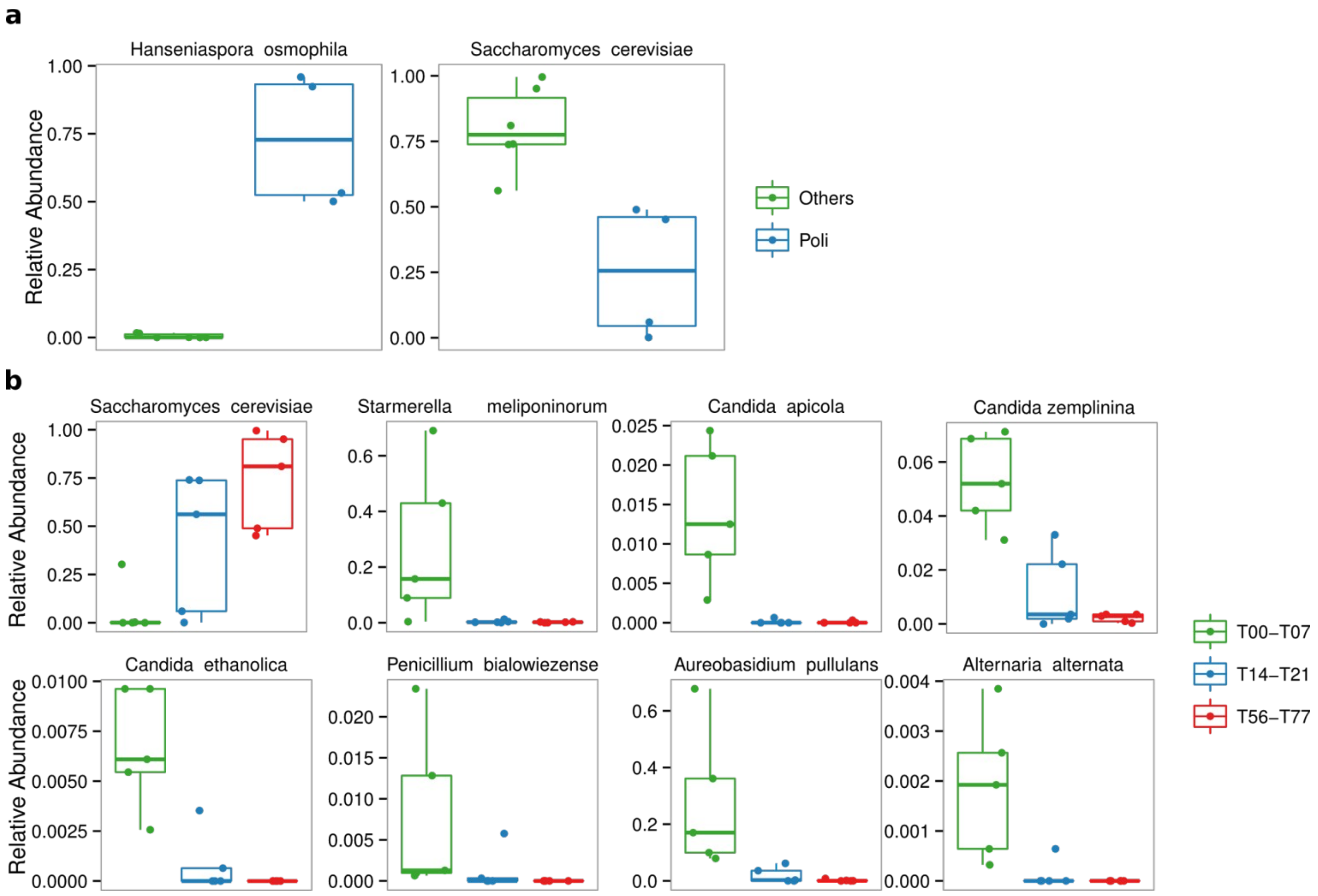

<u>Figure S4:</u> Relative abundances of the six most abundant fungal genera.

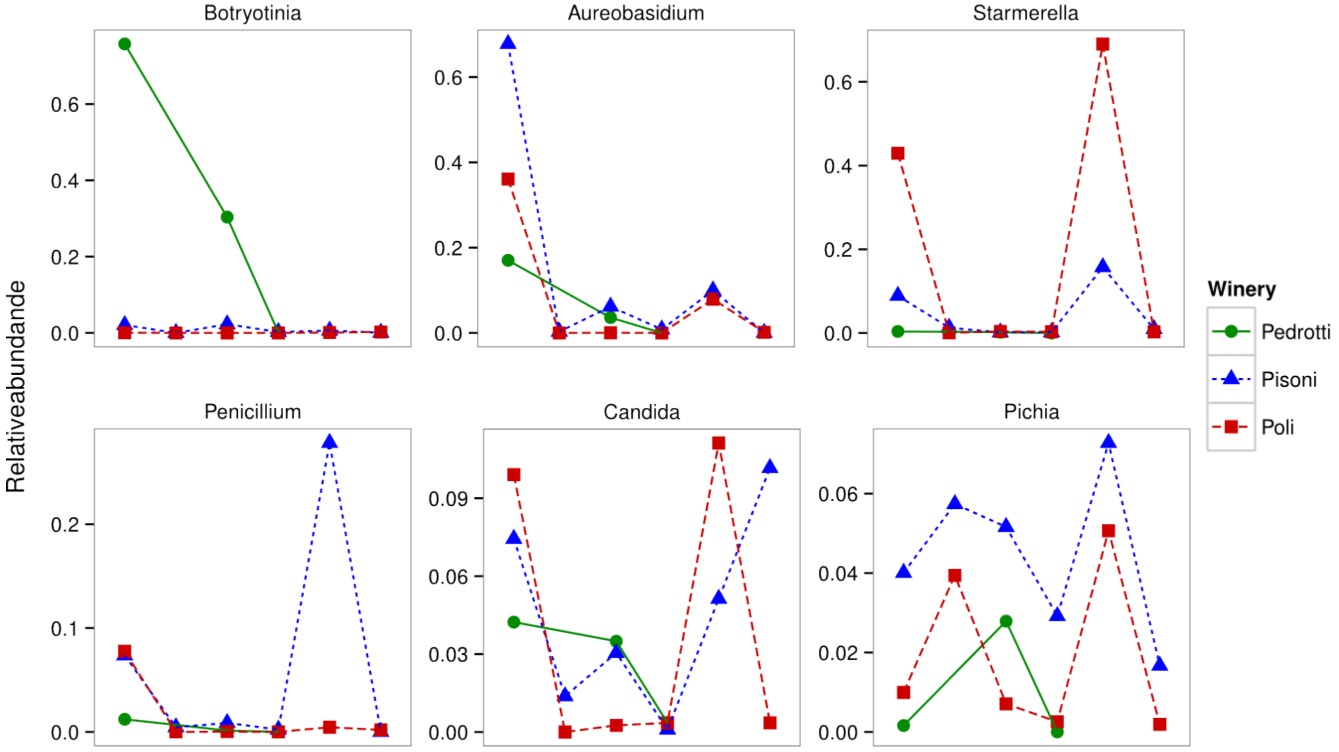

<u>Supplementary Figure 5</u>: PCA of fungal ITS absolute abundance. a- First two principal components of PCA carried out on the absolute abundance of fungal OTUs. According to the PCA components, the loadings of all the fungal species except those indicated in red in the plot (*Saccharomyces cerevisiae* and *Hanseniaspora osmophila*) coincided with the origins of the coordinates (0,0); **b-** *Saccharomyces cerevisiae* absolute representation in the samples studied. **c-** distribution of variables for the first two components of PCA carried out on scaled (zero mean, unit standard deviation) fungal OTU absolute abundance. The loadings of the ten most well-represented fungal species are plotted on the secondary x and y axes.

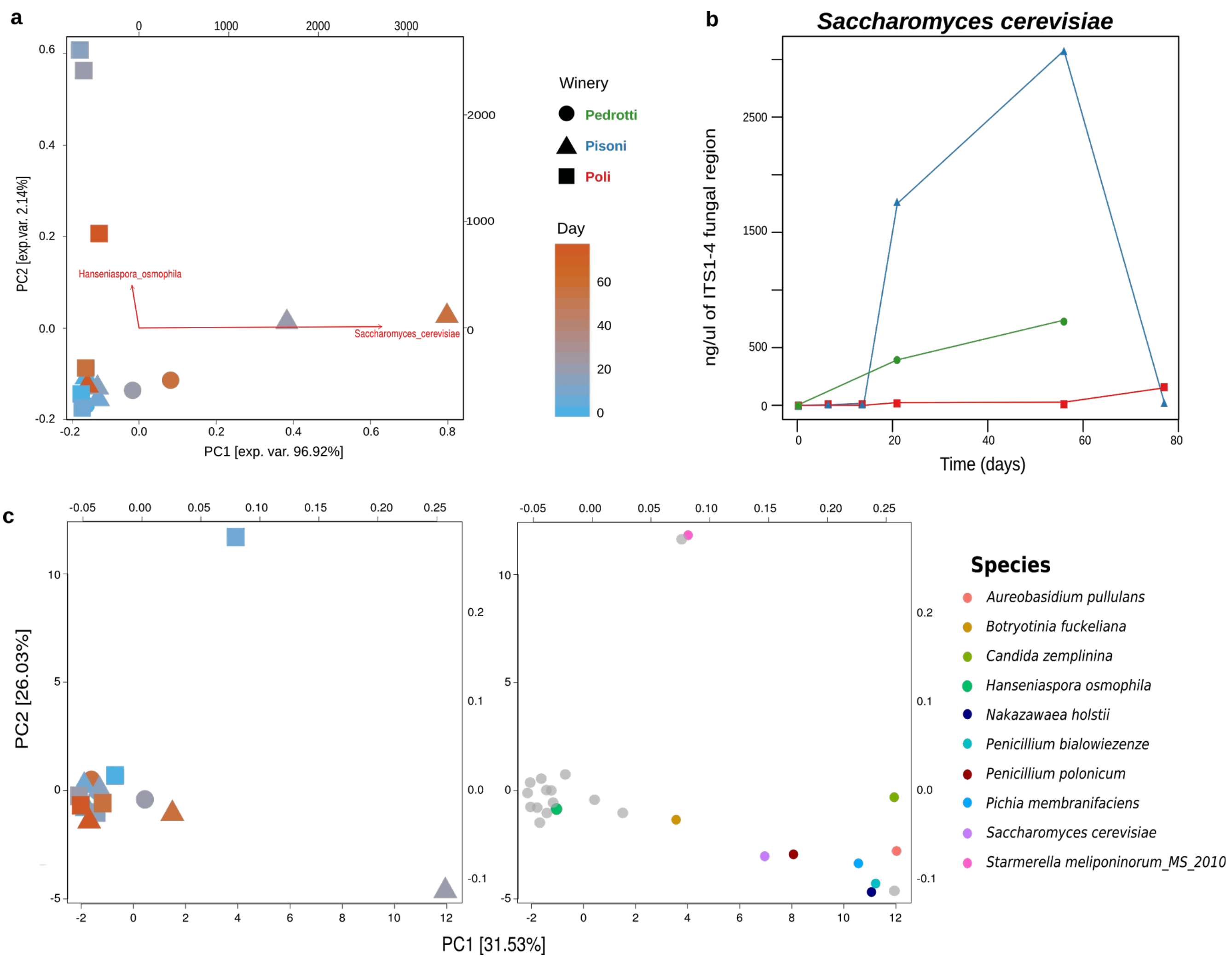

<u>Supplementary Figure 5</u>: Time progression of genera found to be correlated to each other. a- Genera whose relative abundance changes according to the progress of the fermentation process. **b-** Genera whose relative abundance changes independently of the progress of the fermentation process.

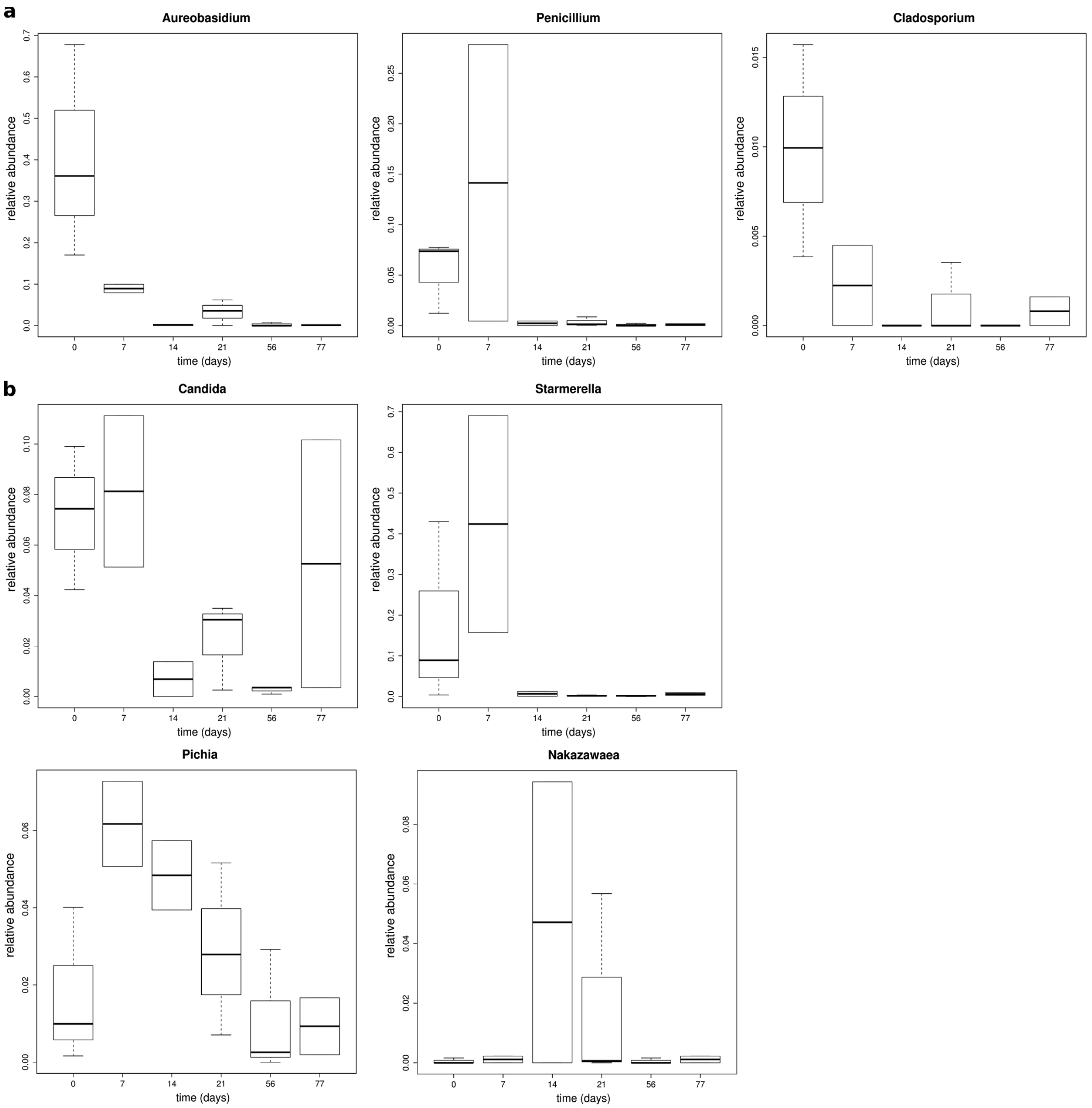

Supplementary Figure 6:

Composition of the *Candida* genus as represented in Vino Santo must during the fermentation process.

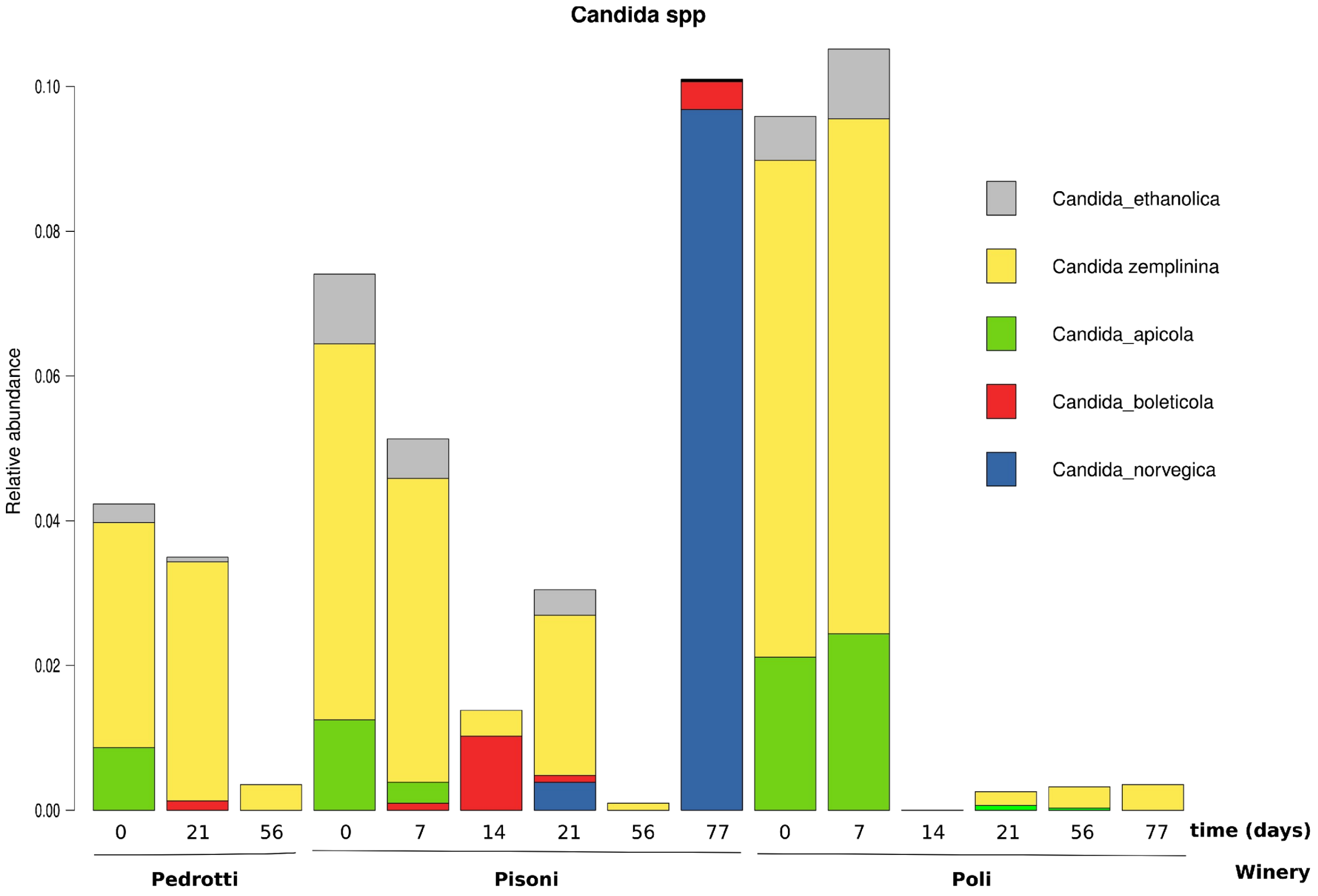

Supplementary Results

Sampling and experimental strategy

To examine the dynamic changes in microbial populations driving must fermentation, three Vino Santo products were investigated from the beginning to the end of fermentation (three months), at the Poli, Pisoni and Pedrotti wineries located in the Valle dei Laghi in Trentino, Italy. The three wineries followed almost the same procedure to carry out Vino Santo fermentation. Vino Santo Trentino is made solely with Nosiola, a grape variety grown almost exclusively in Trentino and in particular in the “Valle dei Laghi” (literally: valley of the lakes). Looser grapes were harvested progressively when they ripened, in our case from late September to mid-October, 2012 and were put on cane trellises to dry until late March, 2013. Traditionally, grapes are crushed during Holy Week (settimana santa), before Easter Sunday. In our case, crushing occurred on 14 March at the Poli winery (must density 32.5° Babo), 2 April at the Pisoni winery (28°Babo), and on 4 April at the Pedrotti winery (36 °Babo). Moreover, no blending was made with other wines. The must was subjected to cold settling only in Pedrotti winery, and no starter culture was added to any of them, so only resident microbiota could grow there. 6,5 g/hL Sulphur dioxide was added to the Poli must, and 7.5 g/hL to the Pisoni must, while the Pedrotti winery added no SO_2 before fermentation. Fermentation occurred in stainless steel tanks, and took place in spring and summer, and stopped naturally when the ethanol concentration reached around 12 - 15 degrees. The wine was then racked and transferred into wood barrels, usually 228 L barriques, where it is aged for 4 to 10 years. Samples were collected weekly from each winery (at least 12 samples for each winery) and named with the number of days passing from the beginning of

fermentation (T0-T88; **Fig. 1a**).

Bacterial populations

Since the fungal population dynamics in must at the Poli vineyard were different from the other two wineries, being driven by both *Saccharomyces cerevisiae* and *Hanseniaspora osmophila*, we investigated the bacterial populations present in these samples.

The number of bacteria increased considerably during the first week of fermentation, then underwent a rapid decrease that continued at a slower rate over subsequent fermentation days, as indicated by qRT-PCR quantification based on the 16S V1-V3 region (**Fig. S7a**). As alcoholic fermentation progressed, the environment became more and more unfavourable to the survival of those bacterial species sensitive to high levels of ethanol. As a consequence, the bacterial populations became less diverse over time (either as described by Chao1 and Shannon diversity indexes or as the number of observed OTUs, **Fig. S8**).

The taxonomic structure of the bacterial component of the microbiota also changed in a time-dependent manner. The relative abundance of Lactic Acid Bacteria (LAB), mainly belonging to the *Oenococcus* genus, increased during the fermentation process until it reached a maximum (30% of the bacterial population) at T21 (**Fig. S9**). As we did for fungi, we explored the existence of correlations between pairs of bacterial OTUs with a relative abundance of over 1%.

Gluconacetobacter, *Acetobacter* and *Gluconobacter* bacterial genera known to play a pivotal role in the structure of microbial populations were found to be positively correlated (**Fig. S7b**). *Oenococcus*, responsible for malolactic fermentation occurring after alcoholic fermentation, was positively correlated with the *Bacillus* genus.

Given that bacteria and fungi can influence each other through either metabolic competition/synergy or

modifications in the environment (Wargo M.J. & Hogan D.A. 2006), we evaluated the existence of correlations between the absolute abundance quantified using qRT-PCR of the ITS and of fungal species and bacterial genera (Spearman correlation). Strong correlations (Spearman’s r=1, P<0.001) were found between the yeasts *Candida ethanolica* and *Pichia fermentans* and the bacteria genera *Hymenobacter*, *Psychrobacterium* and *Ralstonia* (**Fig. S9c**). *C. ethanolica* and *P. fermentans* are known to persist in must during alcoholic fermentation and to produce metabolites contributing to the organoleptic characteristics of the final product (Moreno-Arribas M.V. & Polo C. 2008).

<u>Supplementary Figure 7</u>: Bacteria population during fermentation. a- Fungal and bacterial DNA quantification. Total DNA was quantified through qRT-PCR by using universal primers, amplifying either the V1-V3 16S or the ITS1 region. Standard curves were constructed using PCR products of the V1-V3 16S rDNA or ITS1 regions of a metagenomic sample. **b-** Correlation matrix of bacterial genera. Correlations were calculated on OTU relative abundance. Crossed squares indicate non-significant pairs (P≥0.05). **c-** Correlation networks between fungal and bacterial absolute abundance.

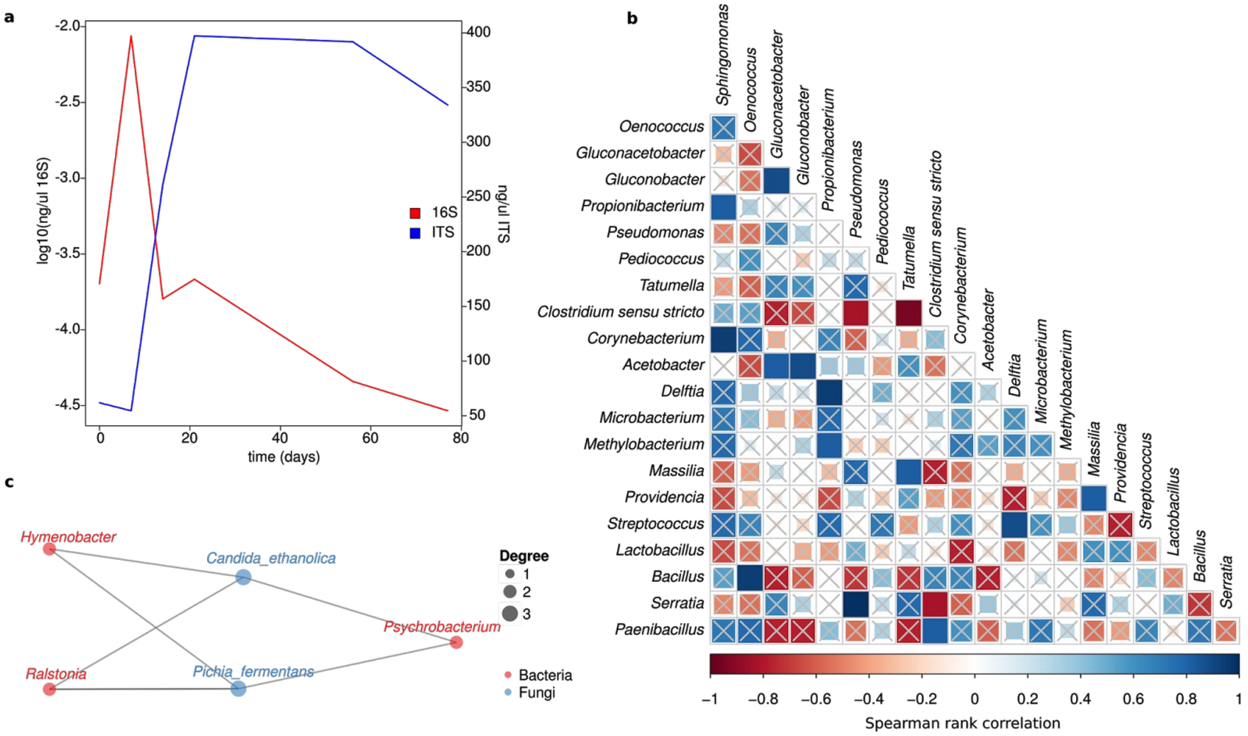

<u>Supplementary Figure 8</u>: Bacterial population alpha diversity estimated according to 3 different indexes.

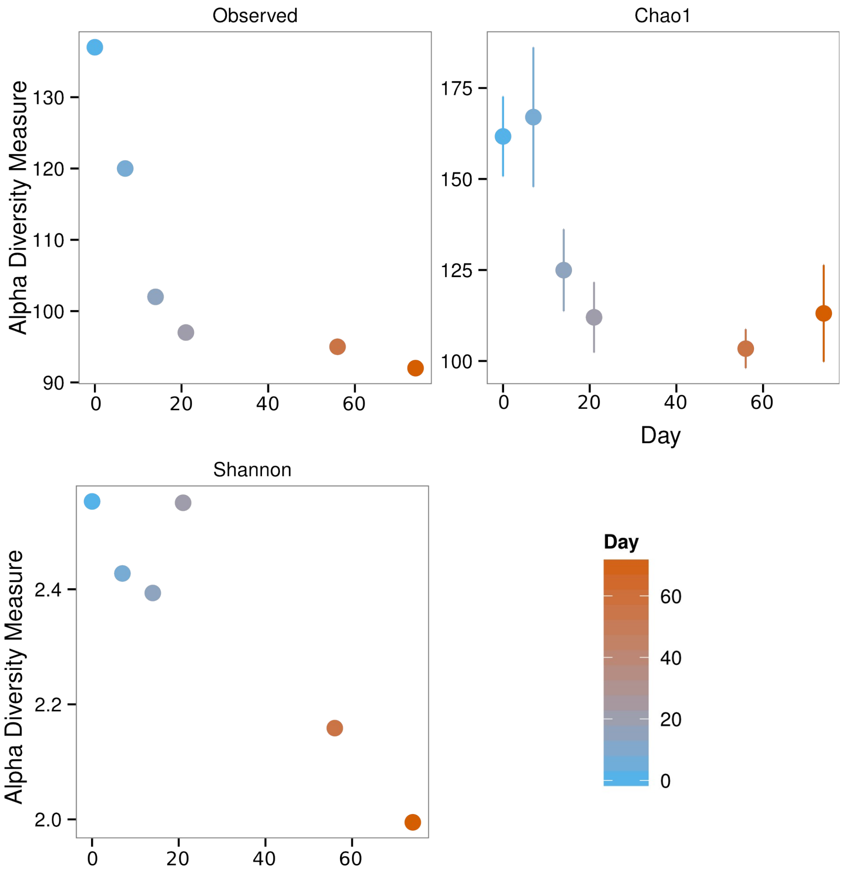

Supplementary Figure 9: Relative abundance of LAB (Lactic Acid Bacteria) over the fermentation period.

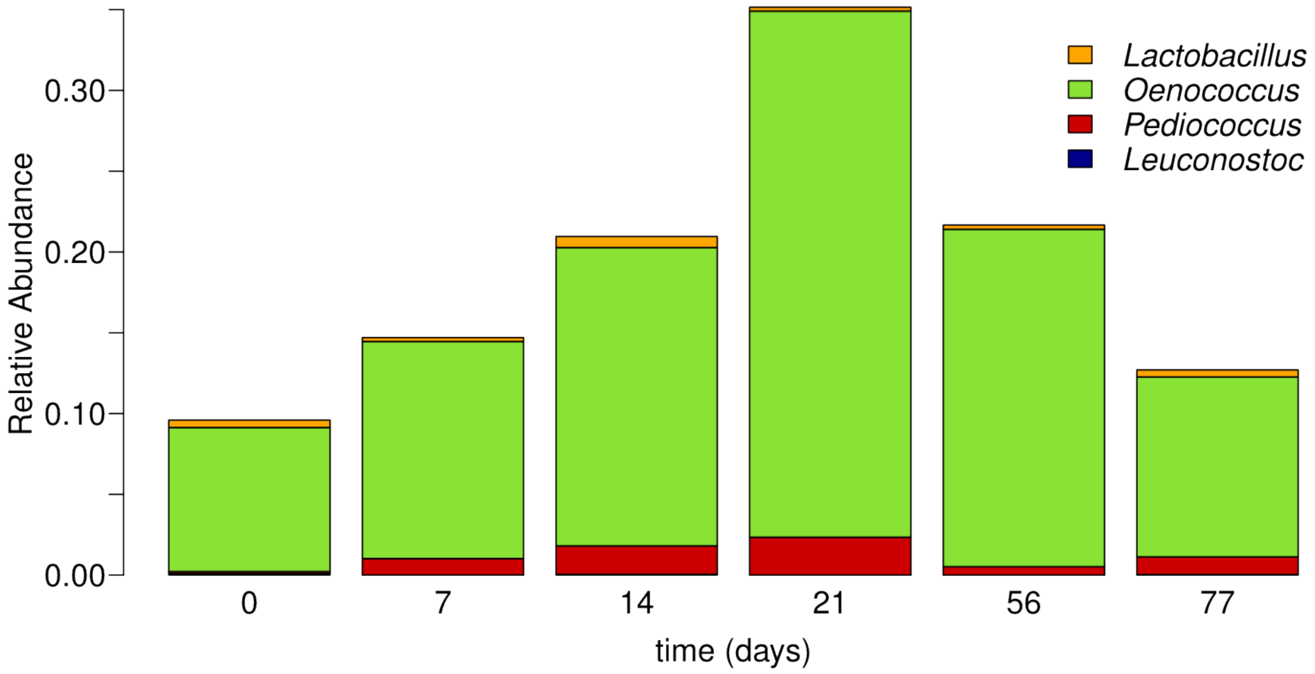

<u>Supplementary Figure 10</u>: Ethanol and glucose concentrations during the fermentation of different Vino Santo musts. **a-** progress of glucose concentration over time. **b-** progress of ethanol concentration over time. **c-** correlation between the total amount of fungi quantified using qRT-PCR in the ITS1 fungal DNA region (black line, mean values for wineries; error bars indicate standard deviations) and the ethanol content of Vino Santo musts at different time points in the three wineries.

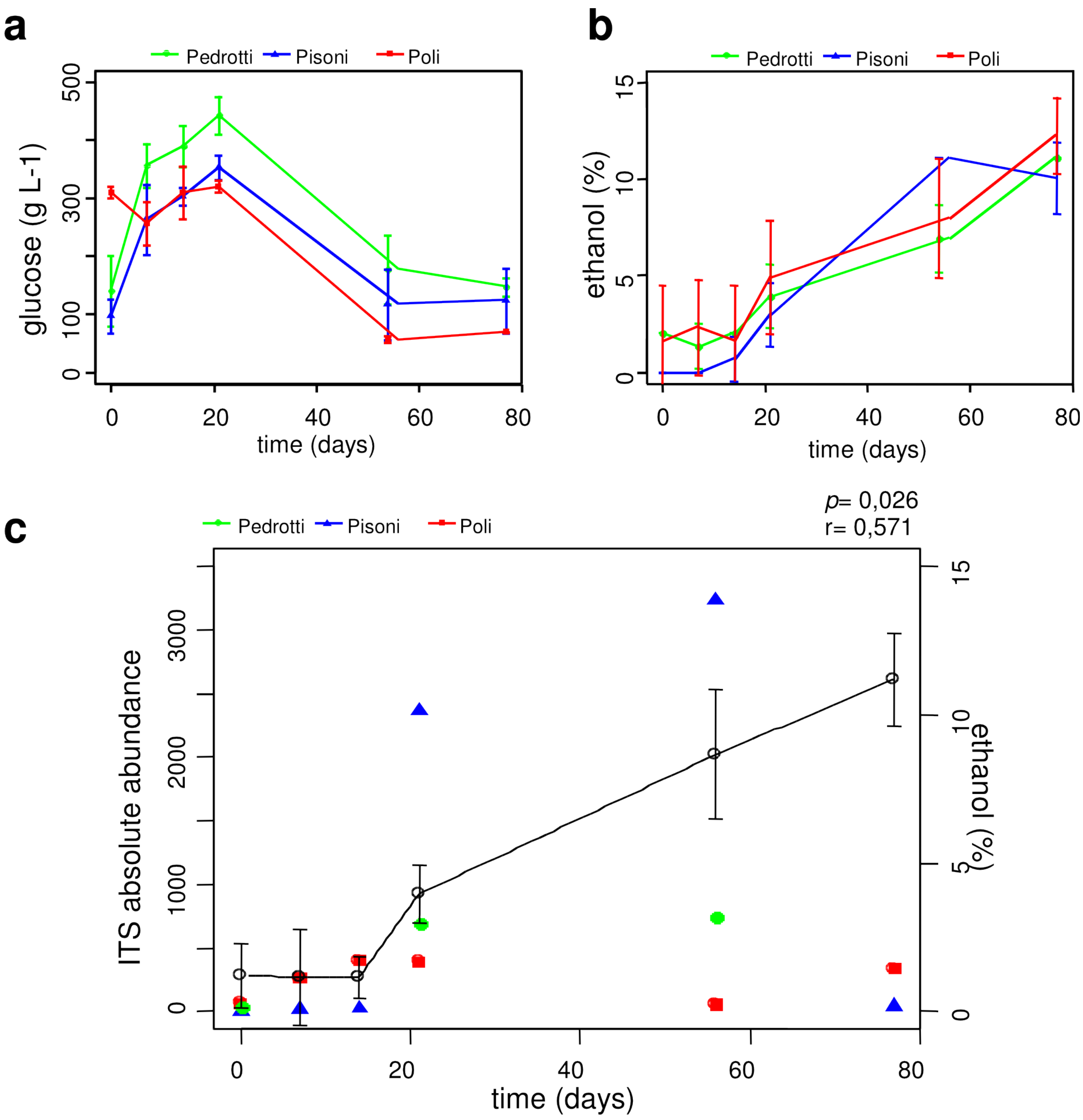

Supplementary Tables

<u>**Supplementary Table S1:**</u> **Summary of samples and analysis.** “x” indicates that the analysis was carried out on the sample. For details on the experimental procedures please refer to the “material and methods” section.

SM “Dynamic changes in microbiota and mycobiota during spontaneous "Vino Santo Trentino" fermentation”

Name	Winery	Day	PCR-RFLP	qRT-PCR	ITS metataxonomics	16S metataxonomics	chemical/physical measurements
PO_T00	Poli	0	x	x	x	x	x
PO_T07	Poli	7	x	x	x	x	x
PO_T14	Poli	14	x	x	x	x	x
PO_T21	Poli	21	x	x	x	x	x
PO_T34	Poli	34	x				
PO_T41	Poli	41	x				
PO_T56	Poli	56	x	x	x	x	x
PO_T61	Poli	61	x				
PO_T67	Poli	67	x				
PO_T77	Poli	77	x	x	x	x	x
PO_T88	Poli	88	x				
PE_T00	Pedrotti	0	x	x	x		x
PE_T07	Pedrotti	7	x				
PE_T14	Pedrotti	14	x	x	x		
PE_T21	Pedrotti	21	x	x	x		x
PE_T27	Pedrotti	27	x				
PE_T34	Pedrotti	34	x				
PE_T41	Pedrotti	41					
PE_T47	Pedrotti	47	x				
PE_T56	Pedrotti	56	x	x	x		x
PE_T61	Pedrotti	61	x				
PE_T67	Pedrotti	67	x				
PE_T77	Pedrotti	77	x				
PE_T82	Pedrotti	82	x				
PE_T88	Pedrotti	88	x				
PI_T00	Pisoni	0	x	x	x		x
PI_T07	Pisoni	7	x	x	x		x
PI_T14	Pisoni	14	x	x	x		x
PI_T21	Pisoni	21	x	x	x		x
PI_T27	Pisoni	27	x				
PI_T34	Pisoni	34	x				
PI_T41	Pisoni	41	x				
PI_T47	Pisoni	47	x				
PI_T56	Pisoni	56	x	x	x		x
PI_T61	Pisoni	61	x				
PI_T67	Pisoni	67	x				

PI_T77	Pisoni	77	x	x	x		x

<u>Supplementary Table 2</u>: Sequences of primers used in this study for various purposes.

Primer Name	Sequence	Purpose
18S-F (forward)	5’-GTAAAAGTCGTAACAAGGTTTC-3’	ITS1 qRT-PCR (fungi)
5.8S-1R (reverse)	5’-GTTCAAAGAYTCGATGATTCAC-3’	ITS1 qRT-PCR (fungi)
F8 (forward)	5’-AGAGTTTGATCMTGGCTCAG-3’	16S V1-V3 pyrosequencing and qRT-PCR (bacteria)
R533 (reverse)	5’-TTACCGCGGCTGCTGGCAC-3’	16S V1-V3 pyrosequencing (bacteria)
ITS1 (forward)	5’-GTTTCCGTAGGTGAACCTGC-3’	ITS1-5.8S-ITS2 PCR-RFLP and pyrosequencing (fungi); (Findley et al., 2013)
ITS4 (reverse)	TCCTCCGCTTATTGATATGC	ITS1-5.8S-ITS2 PCR-RFLP and pyrosequencing (fungi); (Findley et al., 2013)

<u>Supplementary Table 3</u>: Comparison of taxonomic assignments. The taxonomies of all the representative sequences identified in the samples were further checked by comparing the BLAST results of the taxonomic classification against the RDP classifier (v. 2.8) and by manually blasting each sequence through the NBCI Nucleotide collection database. The taxonomic assignment of the vast majority (77.19%) of sequences was confirmed by all the approaches; these sequences which were not univocally assigned to the same taxon at the species level, still matched at the genus level. To ensure reproducibility and comparability of the results, we did not modify the automatic blast taxonomic assignment, in agreement with the accepted standards in the field. (see .xls file)

<u>Supplementary Table 4</u>: Fungal species characterising either Vino Santo fermentation or fermentation at the three wineries.

	PE	PI	PO	PE	PI	PO	PE	PI	PO
Aureobasidium_pullulans	X	X	X	X					
Candida_zemplinina	X	X	X	X					
Saccharomyces_cerevisiae				X	X		X	X	X
Botryotinia_fuckeliana	X			X					
Penicillium_polonicum	X	X							
Hanseniaspora_osmophila				X		X			X
Pichia_membranifaciens		X		X	X			X	
Hanseniaspora_thailandica				X					
Penicillium_bialowiezense		X							
Starmerella_cf_meliponinorum		X	X						
Nakazawaea_holstii					X				
Candida_apicola			X						

<u>Supplementary Table 5:</u> Summary of the results of stepwise regression analysis carried out on OTU relative abundance (observations) and the principal chemicals measured (variables). FDR adjusted p-values were computed using the Benjamini–Hochberg procedure (Benjamini Y. & Hochberg Y. 1995) (see .xls file)

<u>Supplementary Table 6</u>**: Results of Wilcox signed-rank tests to compare chemical values of must from the three wineries.** The columns designed as “P” indicate the p-values resulting from the Wilcox test after FDR (false discovery rate) correction (FDR adjusted p-values were computed using the Benjamini–Hochberg procedure (Benjamini Y. & Hochberg Y. 1995)).

	Pisoni_vs_Poli		Pedrotti_vs_Poli		Pedrotti_vs_Pisoni	
**Value**	**statistic**	**P**	**statistic**	**P**	**statistic**	**P**
**Free SO2 (mg/l)**	7.5	1	3.5	1	2	0.481
**Total SO2 (mg/l)**	5.5	0.561	2	0.481	2	0.481
**Total Acidity (g/l)**	16	***<u>0.029</u>***	1	0.267	0	0.133
**Volatile Acidity (g/l)**	4	0.309	8	0.133	8	0.1
**Z.g.l**	3	0.2	2	0.533	5	0.8
**TARTARIC ACID (g/l)**	13	0.191	7	0.24	4	1
**MALIC ACID (g/l)**	10	0.686	2	0.533	0	0.133
**Density**	3	0.2	2	0.533	5	0.8
**EtOH%**	13	0.47	9	1	11	0.697
**Glu%**	18	1	10	0.897	12	0.548
**pH**	0	***<u>0.002</u>***	8	0.905	18	***<u>0.024</u>***

<u>Sample data</u>: Correspondence between submitted data IDs and samples. Data have been submitted to the European Nucleotide Archive with the accession number PRJEB7999 (http://www.ebi.ac.uk/ena/data/view/PRJEB7999).

References:

- Benjamini Y, Hochberg Y. 1995. Controlling the false discovery rate: a practical and powerful approach to multiple testing. J. R. Stat. Soc. 57:289–300
- Moreno-Arribas MV, Polo C. 2008. Wine chemistry and biochemistry. Springer New York, New York, NY
- Wargo MJ, Hogan DA. 2006. Fungal-bacterial interactions: a mixed bag of mingling microbes. Curr. Opin. Microbiol. 9:359-364.
